# Supplementary material for: Threads of memory: Reviving the ornament of a dead child at the Neolithic village of Ba`ja (Jordan)
Source: PLoS One. 2023 Aug 2;18(8):e0288075. doi: 10.1371/journal.pone.0288075 (PMC10396020; doi:10.1371/journal.pone.0288075)
Supplement: S1 Appendix — PDF document with text and four figures. (PDF) [file pone.0288075.s001.pdf]

Supplementary Information 1

The excavation: method, conditions, observations and first hypothesis

Hala Alarashi[1,2*], Marion Benz[3]

[1] IMF-CSIC, Barcelona, Spain.

[2] Université Côte d'Azur, CNRS, CEPAM, 06300, Nice, France.

[3] Institute of Near Eastern Archaeology, Free University, Berlin, Germany.

Method and documentation on the field

All the elements of the ornament were precisely documented and sketched layer by layer during the excavation (Fig 1a-c). When the beads started to appear, they were given proper numbers, and their position was drawn and documented individually using a cartesian coordination system (x, y, z). This method was applicable insofar the beads appeared in few numbers, scattered, and isolated. The discovery was, however, out of our expectations; as we continued the excavation, the beads appeared in significantly increased numbers, found in concentrations and organised small groups, which required a readjustment of the method of documentation due to time constraints. Therefore, the beads were described, sketched and photographed in small groups, while respecting as much as possible their positions and alignments by introducing, very gently and carefully small pins (Fig 1d, Fig 2f, g, n, o) Otherwise, it would have been impossible to preserve these organisations due to the very loose sand, in which the child was interred. All the bead layers were sketched except the last one (Fig. 1d), discovered after the removal of almost all the bones, for which we had no time left. It was however abundantly photographed and labelled by area and alignments of beads within the area.

Conditions

It was necessary to quickly dig the burial to avoid the deterioration of the skeletal remains and the finds. Yet, the excavation lasted one week (over 84 hours of excavation, documentation, removal and field labeling of the beads) during which the work became a challenge of patience and physical endurance for several reasons: the far access to the area of the beads laying at the bottom, at more than 60cm deep from the upper level of the grave (Fig 2d); the impossibility to excavate from the eastern, northern, or southern sides of the burial because of the surrounding walls. The only access was from the west, laying on the body (Fig 3); stretching the arms, with one hand supporting the weight of the body, while the other excavating; the instability of the beads laying on sandy, extremely loose sediments (To these should be added the heat after 10 a.m. and the endless "curiosity" of tens of flies stuck to our skins).

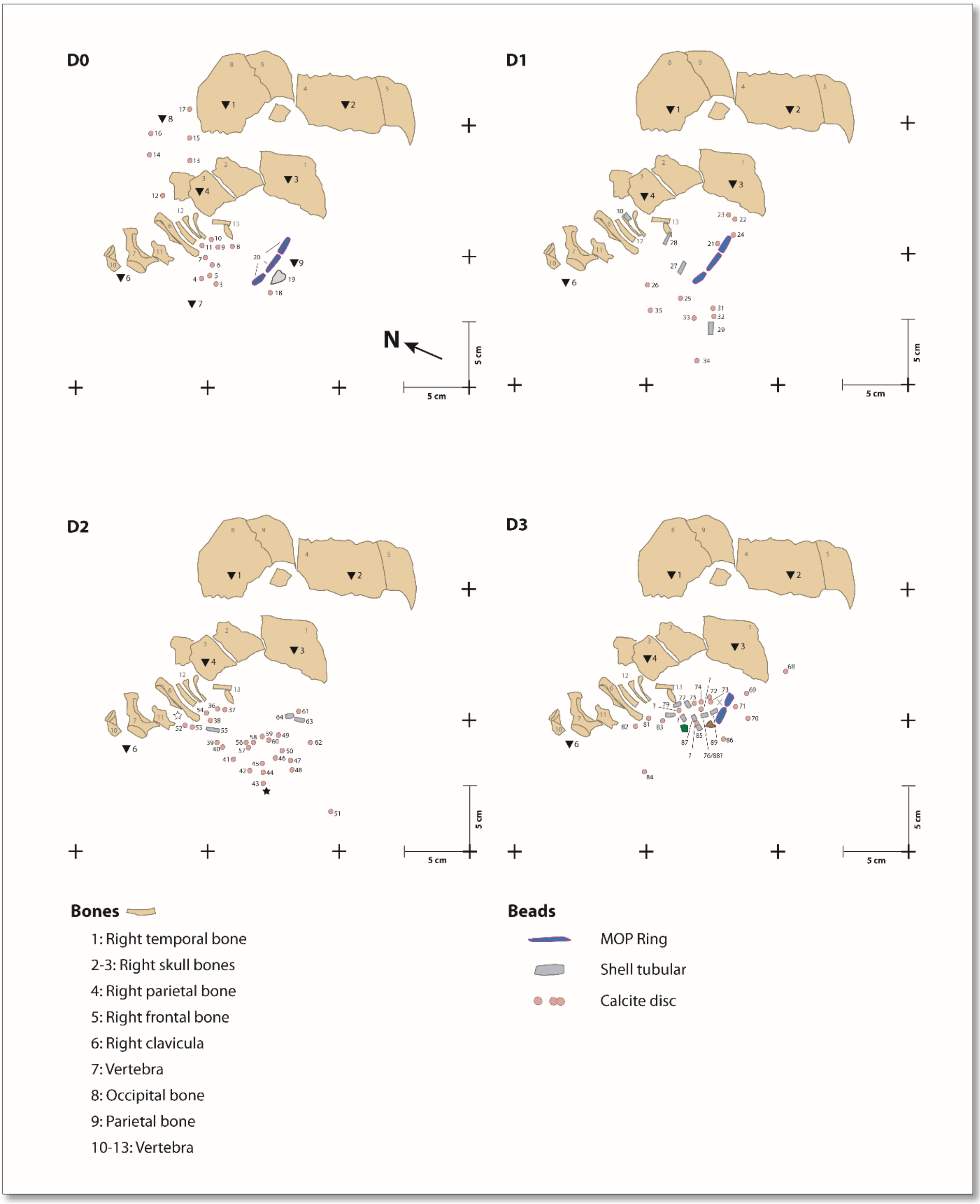

Fig 1a. Sketches showing the distributions of beads, one by one, in relation to the skeletal remains of the child. The depth of layers from D0 to D3 is between 2 and 6 cm.

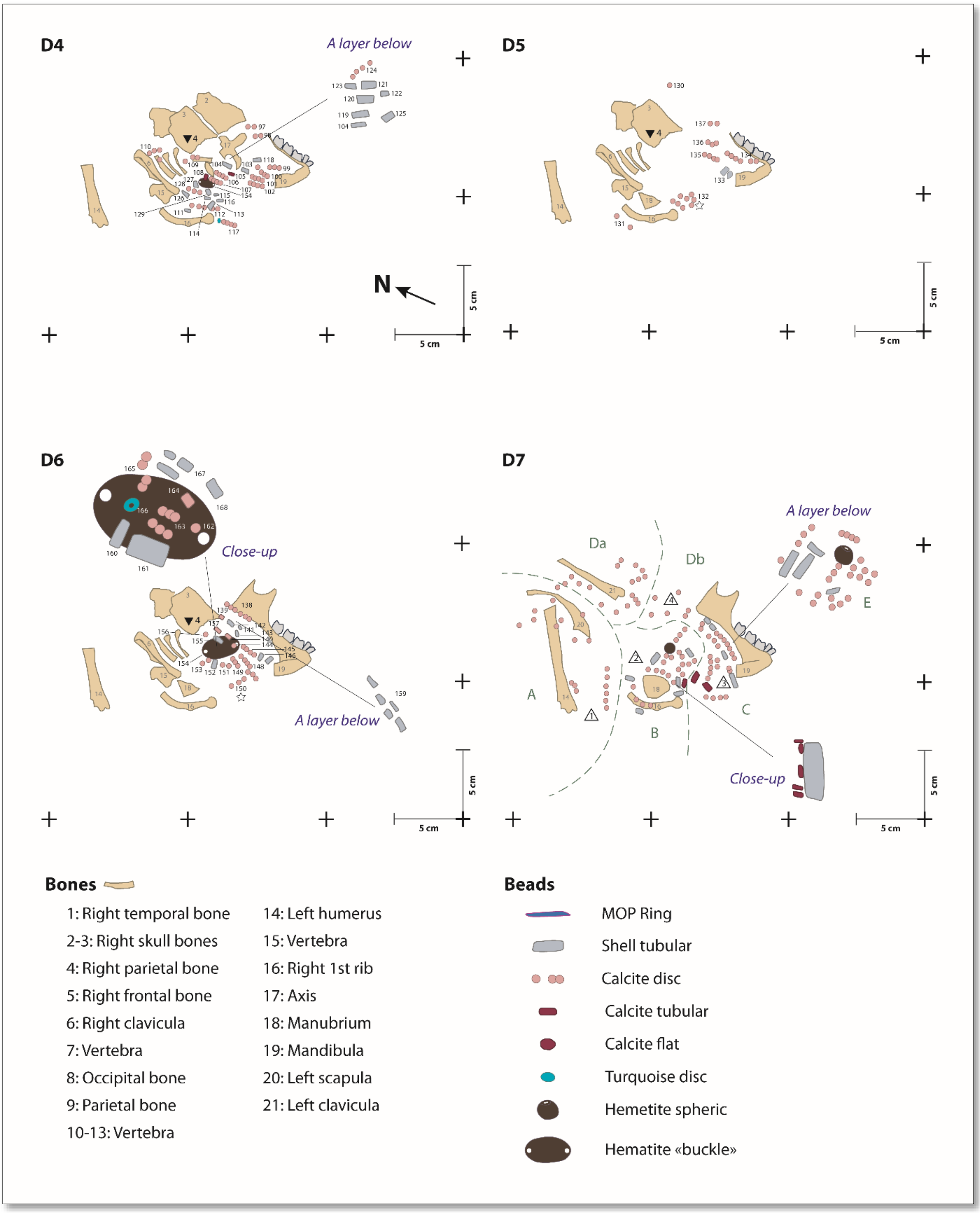

Fig 1b. Sketches D4 to D7 showing the distributions of beads, one by one, in relation to the skeletal remains of the child. From D8 onward, the locations and distribution of beads was made by group and alignment of beads within each group. The depth of layers from D4 to D7 is between 6 and 12 cm.

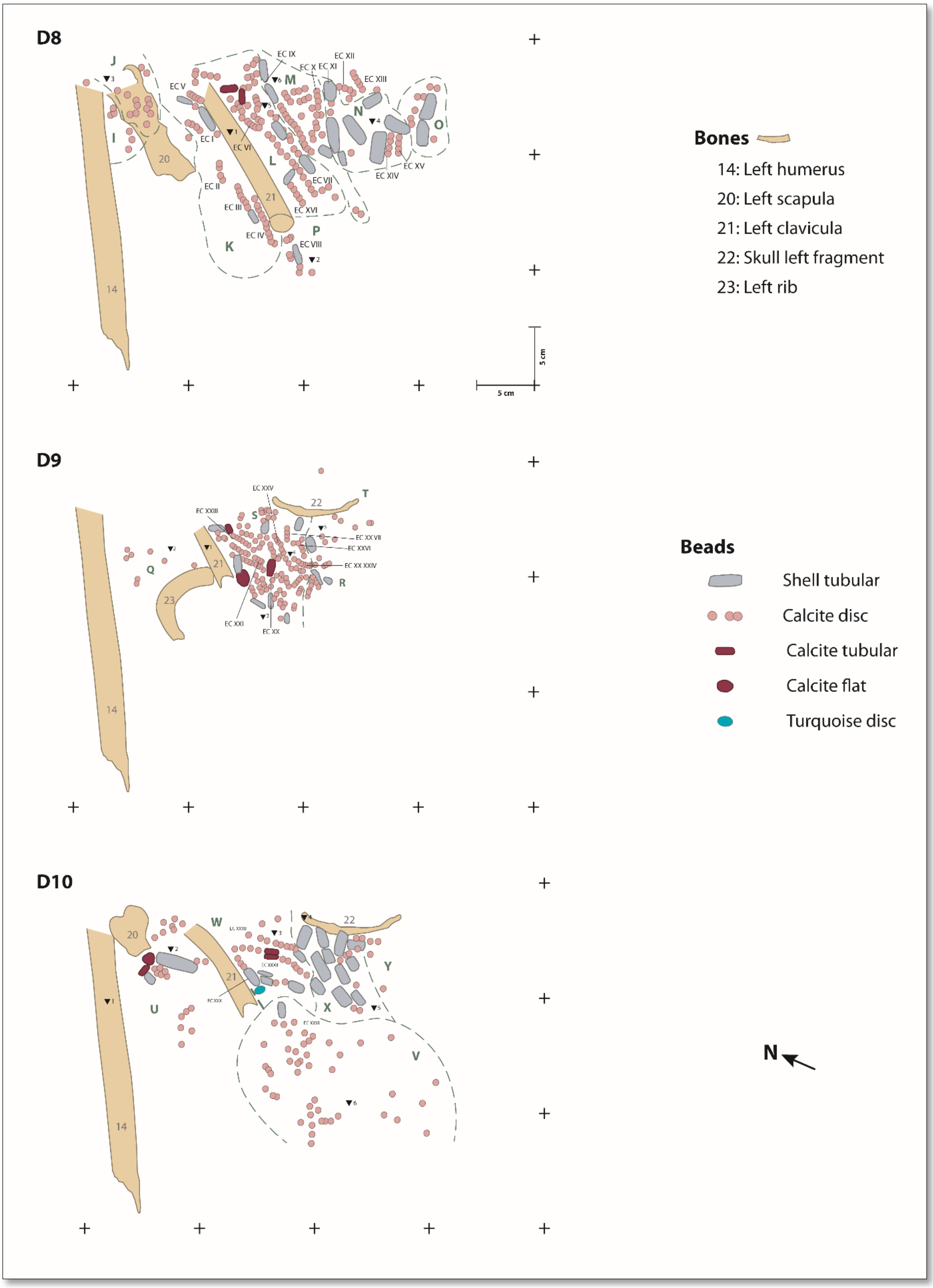

Fig 1c. Sketches showing the distributions of groups and alignments of beads in relation to the skeletal remains of the left part of the upper body. The depth of layers from D8 to D10 is between 3 and 13 cm.

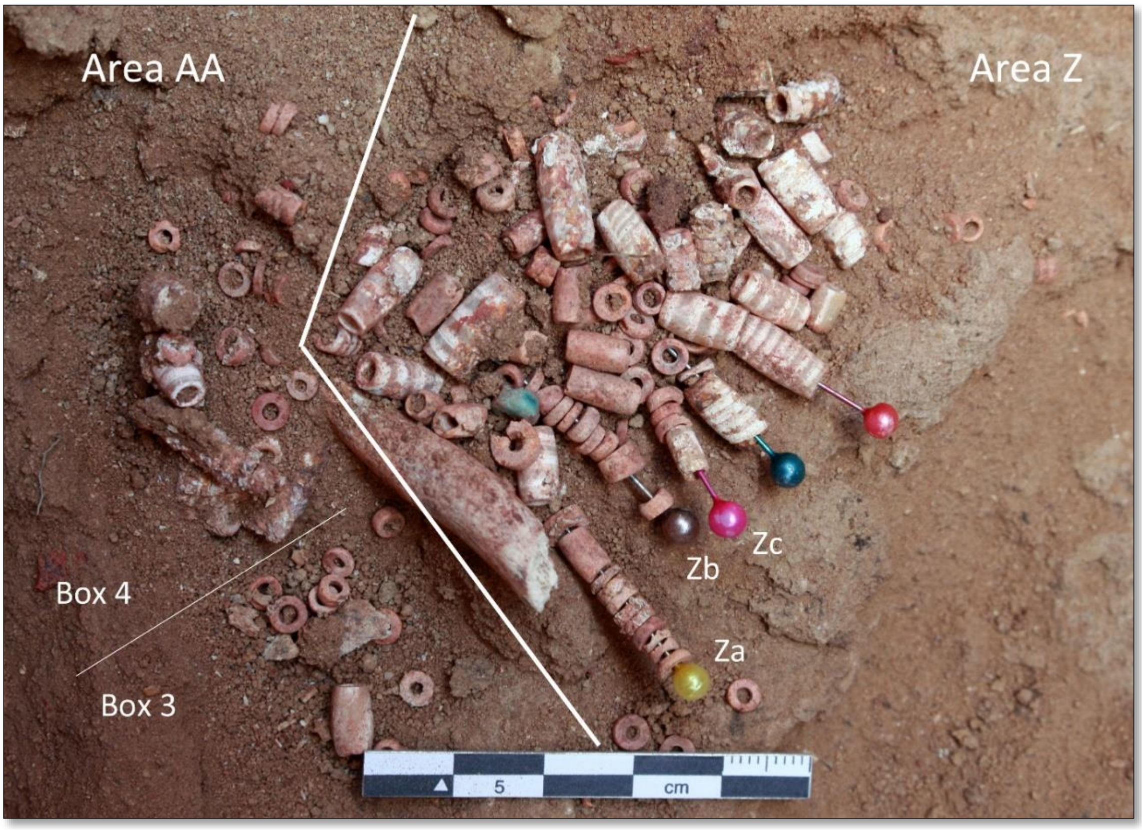

Fig 1d. The last layer of beads subdivided into two areas. Within each, the alignments were documented and labelled. The labels Za, Zb, Zc, Box 3 and Box 4 are an example on the labelling system.

The importance of the first observations

A global understanding of the discovery including the association beads/bones, the dynamics and densities of the excavated layers, and type/colour arrangements of the objects was provided through series of essential observations made during the excavation:

- ✓ The right side of the skeleton was damaged with some bones of the upper layers having completely vanished. The beads associated to the right side of the neck and the ribs (Fig 2e, f; Fig 4a-c), which were the first to be excavated, were scattered around the upper body, showing no clear association or organisation. The density increased, and alignments of beads appeared down towards the left side of the skeleton, while continuing the excavation (Fig 1a-d; Fig 2n; o). Concentrations of beads were also discovered below the left side (*clavicula, scapula*) of the upper body, i.e., the lowest layers of the skeleton, once these had been removed from the silty sand (Fig 1d).
- ✓ None of the beads was found under the arms, on the lateral side of the torso or on the back along the thoracis or the lumbar vertebrae. The elements were strictly distributed on the upper part of the chest and around the neck (Fig 2e-o).
- ✓ While the disc and tubular beads were found almost everywhere, the mother-of-pearl ring and the black "buckle" (Fig 2h) were found in a central position, very close to the

cervical vertebra and behind it respectively. Some beads still stuck to the perforations of these two pieces (Fig 2g, i).

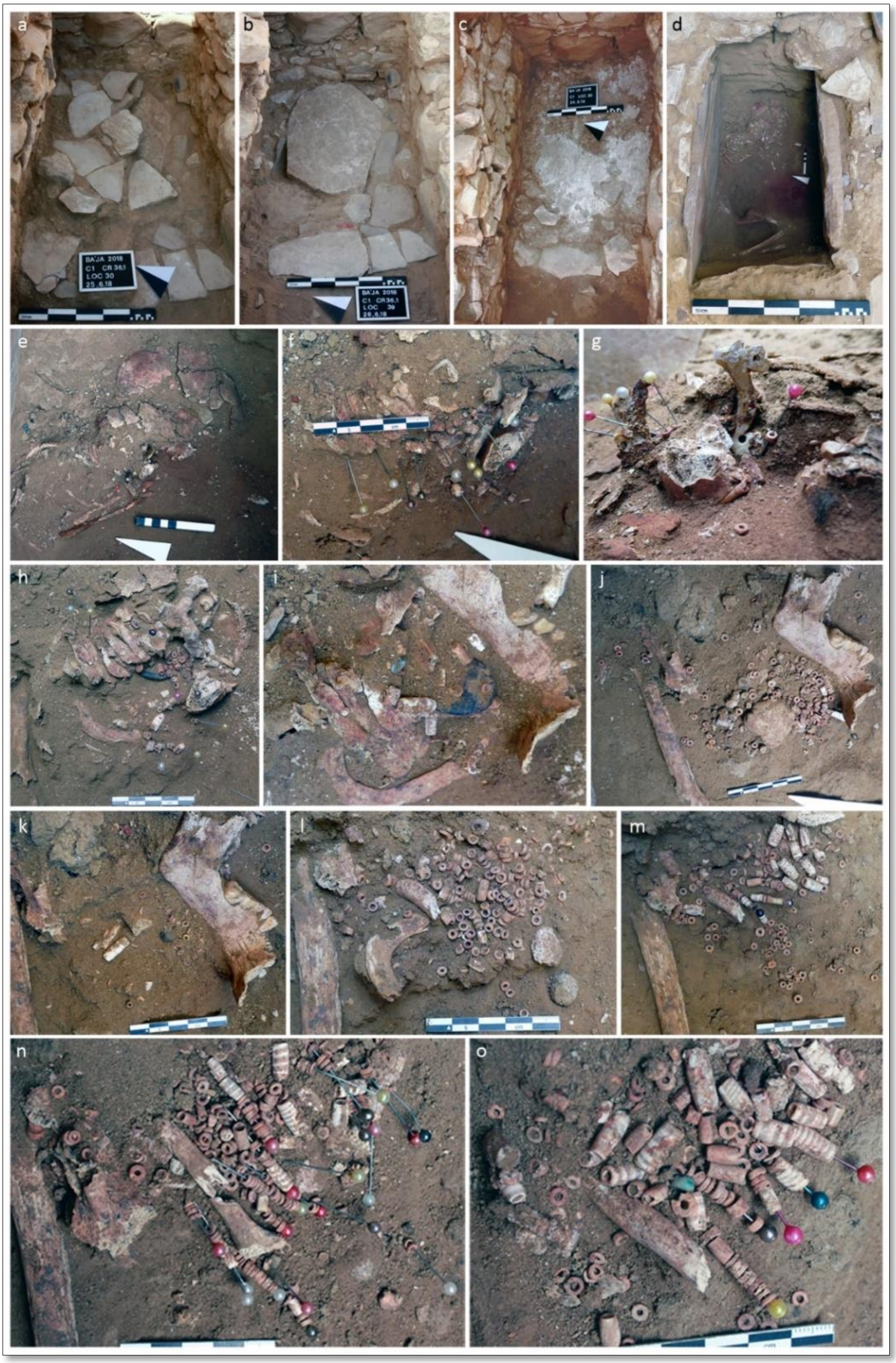

Fig 2. The cist-type burial, the child and the progress of the excavation of the associated ornamental items: a-c the burial sealed with different types and layers of sandstone slabs; d the skeletal remains of the child inside the opened burial; e-f the appearance of the ring around the neck area with scattered little concentrated groups of beads; g view of the ring in its vertical position; h increased density of the beads after the removal of the ring and the appearance of a black element below the cervical vertebra; i beads stuck to the perforation of the black element and appearance of an additional turquoise bead (note the fragmentation and the displacement of the mandible); j the manubrium and a spherical black bead on its upper left; k another spherical black bead appeared just after the removal of the manubrium, placed at its upper right; l-o increased density of the beads in alignments in the area between the left clavicula and the left mandible (removed). (Photos: a-c, e, n, o: M. Benz; d, f-m: H. Alarashi, Ba`ja N.P).

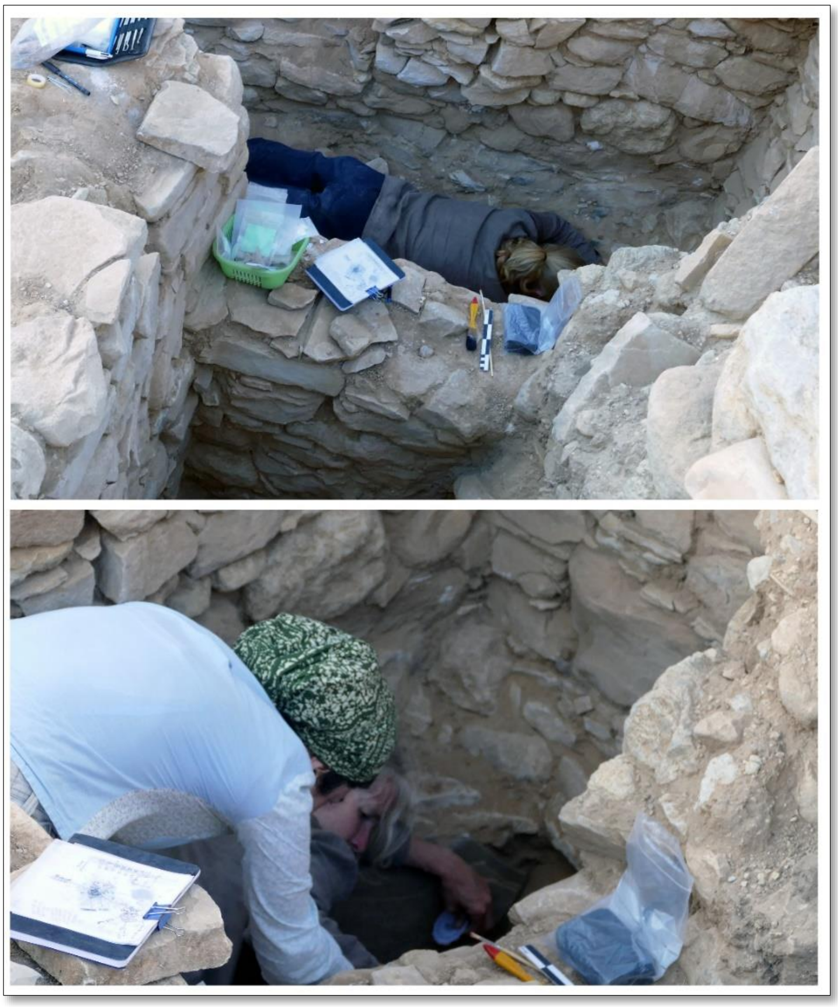

Fig 3. Challenging excavation due to severe space constraints. Marion Benz (upper photo), with Hala Alarashi handling tools (lower photo). (Photos: H.G.K. Gebel, Ba`ja N.P.).

- ✓ Disc beads were distributed all over the area of the neck and chest. Some isolated beads were also present in unexpected places, such as behind the skull near the occipital bones, or the facial bones. It seems that they have been rolled down from their initial position after the decomposition of the string or moved by other taphonomic processes and disturbances by bioturbation.

- ✓ The skull was damaged and fragmented (Fig 1a-c, Fig 2d-e, Fig 4a; c), with the right side of the mandible being decayed and the teeth scattered. A molar was found very close to the mother-of-pearl ring (Fig 4b).
- ✓ The mother-of-pearl ring was found in a vertical position on its edge (Fig 2g). Its upmost damaged edge (Fig 4a, b) appeared when the first beads were unearthed (Fig 4a).

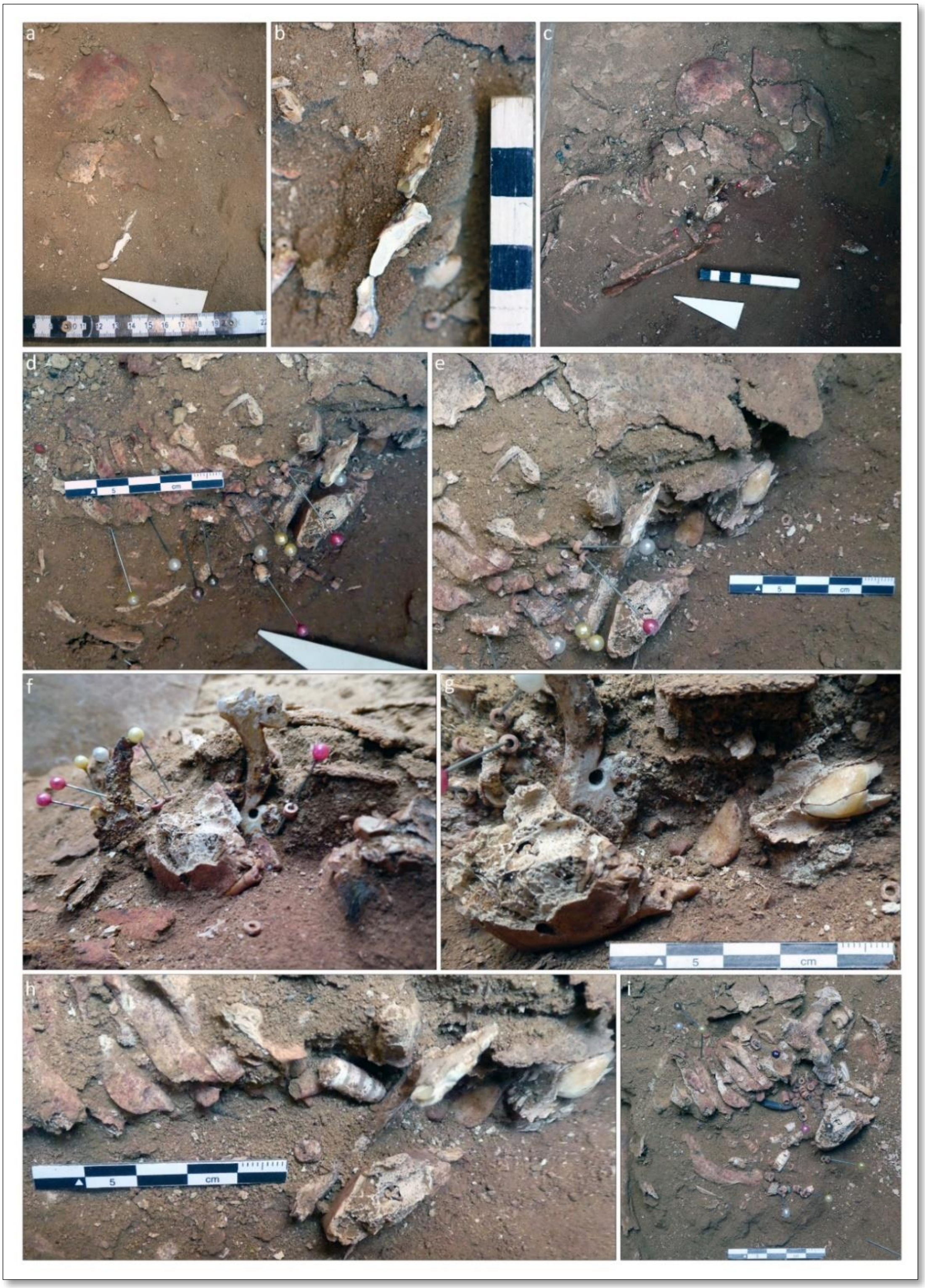

Fig 4. The excavation of the ornament with a focus on the mother-of-pearl ring: a the upmost part of the ring; b with its edge very damaged; c the location of the ring in the area of the neck, between the fragmented skull and the chest; d-e the ring with the first aligned disc bead (note the poorly preserved, broken right mandible just to its right); f-g a vertical view of the ring showing shaped outgrowths around its major diameter; h the lowest edge of the ring reached, with one large tubular bead near one of its perforations; i concentrations of beads appearing after the removal of the ring. (Photos: a-c: M. Benz; d-i: H. Alarashi).

- ✓ The number of beads increased while reaching the bottom of the ring (Fig 2d-e, 4a-c). It was necessary to completely uncover the ring (Fig 4d-h) and remove it to notice that it was laying on the left mandible. This position most likely due to gravity occurring when the child was laid down in the grave. Additionally, the mandible was displaced after the decaying of flesh and ligaments and the ring slipped into it.
- ✓ The "buckle" was discovered under the left side of the cervical vertebra (Fig 2h, i) and next to the ring. Both objects seem to have moved from their initial position, due to gravity as the corpse was resting on its left side.
- ✓ One compact spherical bead was found between the neck and the *manubrium* (slightly to the east of the *manubrium*) (Fig 2j), while another similar one was found at a lower position close to the left side of this bone (on the right side in the photo) (Fig 1k).
- ✓ Groups of tens of beads were found tightly aligned in a chain-like organization, and some remained stuck in this position (Fig 1d; Fig 2h-o). The alignments occurred at several depths and in distinct parts of the distribution area.
- ✓ Tubular white beads were more concentrated in the area corresponding to the left clavicula and the left side of the cervical spine (Fig 2m-o, Fig 4c). They tend to be aligned in groups of two, preceded and followed by several series of red disc beads.
- ✓ Rare objects such as the turquoise beads were found in different parts of the ornament area, some within concentrations or groups of aligned beads, yet rather on the front side of the neck or the chest (Fig 2i, o).

Hypothesis

Based on these observations, several hypotheses, discussed in the results' section, were formulated:

- ➔ The beads, the mother-of-pearl ring, and the "buckle" were part of a composition displayed around the neck and on the chest of the child.
- ➔ The beads were not sewn individually to a garment or a cloth, but threaded on strings to compose several rows, as suggested by the connection of beads with the ring and the "buckle", and by the alignments of beads distributed in several places. This was also confirmed by the use-wear analyses, described in detail in the results section, as several beads show frictional traces diagnostic of bead-to-bead chain-like alignments.
- ➔ The mother-of-pearl ring played a key role in structuring the ornament because of its central position within the area of concentration/ dispersal of the beads, but also because of its uniqueness, large size, decorative features, and the number of its perforations distributed all around its circumference. It is therefore reasonable to

think that it was intended to be displayed on the chest, or even on the tummy, as these positions allow better spreading of the rows of beads, a better display of the beads' diversity, and better visibility of the ring itself

- ➔ The orientation of the ring was determinant for the estimation of the number of rows and for understanding the way the ornament was planned to be exhibited on the body.
- ➔ The position of the body in the grave placed on the left side explains the density of concentration of beads on the left side, as part of those from the right side may have fallen on them after the decomposition of the organic tissues (strings and body of the child). An important number of beads has been displaced and mixed with others.
- ➔ The turquoise and hematite beads were placed in special frontal locations in order to enhance their visibility and to accomplish specific functionalities.
