# Supplementary material for: Threads of memory: Reviving the ornament of a dead child at the Neolithic village of Ba`ja (Jordan)
Source: PLoS One. 2023 Aug 2;18(8):e0288075. doi: 10.1371/journal.pone.0288075 (PMC10396020; doi:10.1371/journal.pone.0288075)
Supplement: S2 Appendix — PDF document of four parts: Palaeoproteomic (Part I), Infrared spectroscopy (Part II), CT-scan (Part III), Morphometric specificities for the study of disc beads (part IV). (PDF) [file pone.0288075.s002.pdf]

Supplementary Information 2

Details on the applied methods

Palaeoproteomic (Part I), Infrared spectroscopy (Part II), CT-scan (Part III) and morphometric specificities for the study of disc beads (part IV)

Part I: Palaeoproteomic analysis of three shell beads

Jorune Sakalauskaite[1,2], Beatrice Demarchi[3], Meaghan Mackie[1,3,4], Matthew Collins[1,4]

[1] Section for GeoBiology, GLOBE Institute, Faculty of Health and Medical Science, University of Copenhagen, Øster Farimagsgade 5, 1353 Copenhagen, Denmark.

[2] Institute of Bioscience, Life Sciences Centre, Vilnius University, Sauletekio ave 7, 10257 Vilnius, Lithuania.

[3] Department of Life Sciences and Systems Biology, University of Turin, Via Accademia Albertina 13, 10123 Turin, Italy.

[4] School of Archaeology, Newman Building, University College Dublin, Belfield, Dublin 4, Ireland

[5] McDonald Institute for Archaeological Research, University of Cambridge

Our analyses unequivocally show that the raw material used to make the three beads among those described and illustrated in the main text (cf. for example figs. 2i-k; fig. 3e) were *Tridacna* sp. shells. Using palaeoproteomics we analysed intracrystalline shell proteins that were extracted from the small archaeological samples. The taxonomic identification was achieved by searching the raw data obtained from high-resolution tandem mass spectrometry against our 'in-house' molluscan protein database, the largest assembled so far. The identified ancient proteins showed features consistent with shell proteins (including domains that are involved in biomineralization processes such as chitin-binding, vWFA, disordered regions). Some peptides had diagenesis related post translational modifications, supporting the endogeneity of the sequences. Dating back to the end of the 8th and the beginning of the 7th mill. cal BCE and coming from the very warm environment at Ba`ja, these represent the oldest molluscan shell protein sequences ever recovered.

Materials and Methods

Protein extraction

Three archaeological samples were selected for palaeoproteomic analyses. The protein extraction procedure followed a method previously described (Sakalauskaite et al. 2019) with few amendments, i.e., the bleaching step was stronger (to isolate the intracrystalline protein fraction) and the purification step was modified in order to retrieve both peptides generated

by proteolytic cleavage and by natural diagenesis (considering the age and the environment where the samples were recovered). Sample details are reported in table 1.

Sample name	Sample name (Turin lab)	Sample weight (mg)	Subsample for analysis (mg)	NaOCl 6-14% (mL)	EDTA (µL)
BJ01	PALTO499	25.5	25.5 (all)	1	510
BJ02	PALTO500	63.4	28.5	1.5	570
BJ03	PALTO501	72.4	37.2	1.5	744
	Blank	-	-	1	150

Table 1. Samples selected for palaeoproteomic analyses. The table reports the names of the samples, their mass (total and used for the extraction) and the volume of solutions used for bleaching and extraction.

The samples were grinded with a micropestle directly in an eppendorf to obtain a fine- grained powder. To obtain the intracrystalline protein fraction, the samples were bleached by adding concentrated NaOCl (6-14%); a blank sample was also prepared. Samples were submerged in bleach for 48 h (periodic agitation in order to ensure mixing) and then removed by centrifugation (10 min, 15k rpm). Samples were thoroughly rinsed with ultrapure water (5x times) and once with ethanol (u.p. grade), before being dried at room temperature (RT). Bleach powders were demineralised with EDTA solution (0.5 M, Sigma-Aldrich, pH=8) in a shaker (1500 rpm, RT) using a ratio of 1:20 (mg/µL, powder in solution). The demineralised samples were reduced using 1M DL-Dithiothreitol (Sigma) for 1h at 65 °C and alkylated with 0.5 M iodoacetamide at RT in the dark. Following this, the samples were centrifuged (10 min, 15k rpm) to separate soluble and insoluble fractions. The two fractions were treated separately. 1) The insoluble fraction was digested with the enzyme trypsin. 2) The soluble fraction was processed without digestion, using a protocol described for ancient enamel samples (Welker et al. 2020), but adapted here to shells, considering that natural diagenesis may have already cleaved up some proteins and peptides. The different fractions were recombined at the peptide clean-up step. Treatment procedure in detail: 1) to all the insoluble aliquots, 100 µL of Ambic solution was added (50 mM, pH 7.5–8) and digestion carried out with 1 µL of trypsin (0.4 µg/µL, Promega, proteomics grade), overnight (~16 hrs). Digestion was stopped with 1 µL of 10% TFA. The digests were further acidified with 10 % TFA to reach pH ≈2 before the peptide clean-up with C18 stage tips. 2) The soluble aliquots were directly acidified to lower down the pH to ≈3-4 using TFA (10% and 50%). The peptides obtained from the soluble and insoluble fractions were purified and combined using in-house made C18 stage-tips (Rappsilber et al. 2007). The C18 tips were equilibrated and washed according to standard protocol. The soluble fraction was loaded onto the C18 resin, then washed with 0.1%

TFA solution to remove any leftovers of salt, before the peptides from the insoluble fraction were loaded to the same tips. The tip was washed again with 0.1 % TFA solution and finally eluted twice with 20 µL of each 60% ACN and 40% ACN. The eluate was concentrated in a vacuum centrifuge before LC-MS/MS analysis.

LC-MS/MS analysis

The remaining eluate was resuspended with 0.1% TFA and 5% ACN solution and injected in an EASY-nLC 1200 (Thermo Fisher Scientific, Bremen, Germany) coupled to a Exploris 480 orbitrap mass spectrometer (Thermo Fisher Scientific, Bremen, Germany) on a 77 min gradient. Chromatographic and MS parameters were then performed based on previously published methods for ancient and degraded samples (Mackie et al. 2018), with minor adjustments to account for parameter setting differences between different mass spectrometer versions. MS parameters were set as follows: MS1 – 120,000 resolution at *m/z* 200 over the *m/z* range 350–1400, AGC target of 300, maximum injection time (IT) of 25 ms; MS2 – top 10 mode, 60,000 resolution, AGC target of 200, maximum IT of 118 ms, HCD collision energy of 30% and dynamic exclusion of 20 s.

Bioinformatic search

Creating Molluscan protein database

Bioinformatic analyses of raw data obtained from LC-MS/MS runs were performed using PEAKS Studio 11 software. PEAKS algorithm performs *de novo* sequencing after which peptides can be identified and matched to protein sequences using a database search. For this project, we created a comprehensive Molluscan protein database using sequences present in public databases and publications. The database included two components: 1) Molluscan proteins downloaded directly from NCBI and 2) proteins translated from molluscan transcriptomes. The latter were obtained from different sources: a) by downloading sequences from the TSA (translated shotgun assemblies) repository based in NCBI, b) *de novo* assembled mantle transcriptome for *Tridacna crocea* recently published by (Takeuchi et al. 2021); c) *de novo* mantle transcriptome for *Spondylus gaederopus* which has been extracted and assembled by our group (Sakalauskaite et al., in preparation).

Detailed description of database components: 1) Molluscan protein NCBI dataset was created by downloading all of the sequences from NCBI protein repository, restricting the taxonomy to phylum Mollusca and selecting the classes of bivalves, gastropods and tusk shells. Sequences were downloaded on 08.03.2021 and included in total 297891 sequences (including common laboratory contaminant proteins as these are often observed in analyses). 2). TSA sequences (assembled transcriptomes) were downloaded from NCBI sequence set browser restricting the taxonomy to Mollusca and belonging to either gastropods or bivalves (no transcriptome projects were present for tusk shells, download date 13.04.2021). The sequencing projects were further filtered to retain those where RNA was isolated either from

the mantle or the whole body (i.e., transcripts that have the highest likelihood to be related to shell production). The downloaded sequences were combined and further processed on UseGalaxy Europe platform (Galaxy Community 2022). We also added two *de novo* assembled molluscan mantle transcriptomes from *Tridacna crocea* (Takeuchi et al. 2021) and *Spondylus gaederopus*. The RNA data were translated to protein sequences using the TransDecoder tool which finds coding regions within transcripts. The output fasta files were renamed (to include species name on each protein sequence) and combined to one large protein dataset from transcripts. This accounted for a total of 1463816 sequences from 41 different molluscan species, belonging to 14 different bivalve families (i.e., Ostreidae, Mytilidae, Cardiidae, Spondylidae, Pteriidae, Pectinidae, Unionidae, Pinnidae, Arcidae, Lucinidae, Solecurtidae, Tellinidae, Vesicomyidae, Dreissenidae) and 11 different gastropod families (i.e., Limacinidae, Haliotidae, Helicidae, Naticidae, Littorinidae, Lymnaeidae, Ampullariidae, Planorbidae, Camaenidae, Viviparidae, Semisulcospiridae). Finally, all of the sequences were combined together (from transcripts and proteins). This "Molluscan Protein database" accounts to a total of 1761707 sequences.

Peptide and protein identification

Peptide and protein identification was obtained via Peaks Studio 11 software using Molluscan Protein database described above. Search parameters: semi specific tryptic digestion, fragment ion mass error tolerance 0.05 Da and precursor ion mass error tolerance 10 ppm. For peptide identification and protein match we used the results obtained by SPIDER searches (including all possible modifications). Threshold values for the acceptance of high-quality peptides: false discovery rate (FDR) 1 %, protein scores −10lgP ≥ 30, unique peptides ≥ 2, *de novo* sequences scores (ALC %) ≥ 50. All of the identified protein sequences were checked and assessed manually. Proteins were excluded from further analyses in cases where: a) they were supported only by peptides with low complexity domains (LCDs). These domains are a common feature of many shell proteins (Sakalauskaite et al. 2019) and are not taxon specific; b) supporting peptides and protein showed homology to contaminant sequences (e.g. trypsin, keratin); c) they were also found in blank samples and thus, further considered as contamination. Protein classification and the identification of domains was carried out using the InterPro tool (Blum et al. 2021). BlastP search was used to check the identified sequences if they were homologous to other types of proteins.

After analysing the data with Molluscan protein database, we found that most of the non-LCD-type peptides matched to protein sequences from *Tridacna* sp. (Mollusca, Bivalvia, Cardiida, Cardiidae, *Tridacna*). Therefore, as our next step, we carried out a second protein search with PEAKS software using a database restricted to *Tridacna* proteins (i.e., we gathered all of the sequences that were related to genus *Tridacna*, mainly belonging to the species *T. crocea* and *T. maxima;* this dataset contained 105031 sequences). The use of smaller databases allows us to identify more proteins. We also wanted to see if the observed LCD peptides can be matched to *Tridacna* proteins. For this round of PEAKS search, we used more stringent parameters - the value of −10lgP was set to ≥ 40 (unique peptides ≥2). The list of identified proteins using both databases is presented in Table 2.

Sample	Sequence No	Accession	Organism	InterPro analysis		DB: Molluscan Proteins				DB: Tridacna proteins				BlastP search	PTMs
				Classification	Domains	Coverage (%)	Peptides Total	Peptides Unique	-10lgP score	Coverage (%)	Peptides Total	Peptides Unique	-10lgP score		
P499	1	[Tridacna_crocea]_TRINITY_DN253411_c2_g2_i3.p1	*Tridacna crocea*	Uncharacterized	Disordered	14.09	23	19	242.21	24.38	58	44	398.21	No significant hits	Deamidation, oxidation, dehydration
P500										5.51	6	6	159.6		deamidation, oxidation, pyro-glu from Q
P501						13.14	14	14	197.76	19.60	30	25	358.94		Deamidation, oxidation
P499	2	[Tridacna_crocea]_TRINITY_DN236812_c0_g2_i1.p1	*Tridacna crocea*	Uncharacterized	Disordered	12.05	5	4	134.02	19.68	11	11	206.34	No significant hits	deamidation, Pyro-glu from E
P500										5.62	2	2	74.12		
P501						8.43	5	5	121.50	11.24	8	8	225.2		Deamidation, pyro-glu from E, dehydration
P499	3	[Tridacna_crocea]_TRINITY_DN248626_c100_g1_i2.p1	*Tridacna crocea*	Uncharacterized	Disordered	5.57	5	5	146.29	7.16	9	9	250.92	Uncharacterized proteins	
P500										6.16	9	9	151.43		Oxydation, pyro-glu from E
P501						3.78	3	3	97.13	5.17	6	6	228.73		Oxidation
P499	4	[Tridacna_crocea]_TRINITY_DN256058_c0_g2_i1.p1	*Tridacna crocea*	Chitin-binding protein	Chitin-binding; vWFA; Thrombospondin type-1 (TSP1) repeat	1.28	5	5	98.43	2.56	11	11	193.06	SCO-spondin-like proteins & other uncharacerized mollusc proteins	
P500										5.08	30	30	216.74		Deamidation, oxidation
P501						2.31	11	11	163.52	6.89	44	44	336.75		Deamidation, oxidation
P500	5	[Tridacna_crocea]_TRINITY_DN226875_c1_g4_i1.p1	*Tridacna crocea*	Uncharacterized		32.28	2	2	82.47	71.65	42	42	369.88	No significant hits	
P501						45.67	11	9	172.27	65.35	34	34	331.46		Dehydration, phosphorylation, oxidation
P500	6	[Tridacna_crocea]_TRINITY_DN242293_c0_g1_i2.p1	*Tridacna crocea*	RNA binding protein	Serine/arginine-rich splicing factor SRSF4-like; RNA recognition motif					16.03	15	15	231.34	Serine/arginine-rich splicing factor-like proteins from other molluscs	Phosphorylation
P501										14.89	13	13	178.85		Phosphorylation
P500	7	[Tridacna_crocea]_TRINITY_DN186455_c0_g1_i3.p1	*Tridacna crocea*	Uncharacterized						39.69	21	21	266.26	No significant hits	Dehydration
P501										24.51	8	8	134.96		

P500	8	[Tridacna_crocea]_TRINITY_ DN233716_c0_g1_i6.p1	*Tridacna crocea*	Uncharacterized						29.63	13	13	272.33	No significant hits	Phosphorylation
P501										26.54	10	10	255.98		Phosphorylation
P500	9	[Tridacna_crocea]_TRINITY_ DN256022_c0_g1_i1.p1	*Tridacna crocea*	Mucin-like protein	Chitin-binding; disordered					4.10	17	17	123.35	Mucin-like and Serine-rich adhesin-like proteins from other molluscs	Deamidation, oxidation
P501										3.74	13	12	158.45		Deamidation, oxidation
P500	10	[Tridacna_crocea]_TRINITY_ DN256186_c0_g1_i1.p1	*Tridacna crocea*	Nucleic acid binding protein	RNA-binding motif; DNA-binding site; disordered					6.25	7	7	198.57	Uncharacerized proteins (Symbiodinium sp.)	
P501										6.70	8	8	192.39		Oxidation
P500	11	[Tridacna_crocea]_TRINITY_ DN241046_c51_g11_i1.p1	*Tridacna crocea*	RNA-binding protein	RNA recognition motif; DNA-binding site					4.08	4	4	127.29	Ribonucleoproteins from other molluscs	Deamidation, oxidation
P501										8.15	5	5	84.60		Deamidation, hexose
P499	12	[Tridacna_crocea]_TRINITY_ DN251608_c0_g1_i2.p1	*Tridacna crocea*	Uncharacterized	Disordred	1.07	1	1	396.55	2.59	2	2	60.22	TITIN-like & shematrin proteins from other molluscs	Deamidation
P500										2.59	2	2	81.65		Deamidation
P499	13	[Tridacna_crocea]_TRINITY_ DN253411_c2_g2_i2.p1	*Tridacna crocea*	Uncharacterized	Disordered					16	7	4	181.29	Uncharacterized proteins	Deamidation, oxidation
P499	14	[Tridacna_crocea]_TRINITY_ DN253411_c2_g1_i1.p1	*Tridacna crocea*	Uncharacterized	Disordered	10.4	4	2	136.1					No significant hits	Deamidation
	14a	[Tridacna_crocea]_TRINITY_ DN253411_c2_g1_i3.p1								12.7	8	2	223.9		
P499	15	[Tridacna_crocea]_TRINITY_ DN254257_c1_g18_i4.p1	*Tridacna crocea*	Pleckstrin-like protein	Pleckstrin					2.86	2	2	48.77	Pleckstrin homology-like proteins from other molluscs	
P500	16	[Tridacna_crocea]_TRINITY_ DN151759_c1_g1_i1.p1	*Tridacna crocea*	RNA-binding protein	RNA recognition motif					21.26	6	4	177.98	Ribonuclearproteins from Symbiodinium sp.	
P500	17	[Tridacna_crocea]_TRINITY_ DN188550_c0_g1_i1.p1	*Tridacna crocea*	Uncharacterized	Chromatin target of PRMT1 protein; disordered					10.78	8	8	110.66	Unamed proteins from Symbiodinium sp.	
P500	18	[Tridacna_crocea]_TRINITY_ DN250769_c0_g1_i3.p1	*Tridacna crocea*	RNA-binding protein	RNA recognition motif					13.12	5	5	85.27	Heterogeneous nuclear ribonucleoprotein	Hexose, oxidation, deamidation

														s from other molluscs	
P500	19	[Tridacna_crocea]_TRINITY_ DN253868_c0_g2_i1.p1	*Tridacna crocea*	ATP-dependent RNA helicase	RNA helicase; disordered					6.61	5	5	81.13	ATP-dependent RNA helicase-like proteins from other molluscs	Deamidation
P500	20	[Tridacna_crocea]_TRINITY_ DN254985_c6_g5_i5.p1	*Tridacna crocea*	Uncharacterized	Disordered					3.00	2	2	47.31	No significant hits	
P501	21	[Tridacna_crocea]_TRINITY_ DN252988_c185_g7_i1.p1	*Tridacna crocea*	Uncharacterized						17.86	2	2	120.03	No significant hits	Deamidation
P501	22	[Tridacna_crocea]_TRINITY_ DN256066_c2_g1_i1.p1	*Tridacna crocea*	BSMP-like protein	VWF_A; chitin binding					1.38	3	3	134.04	BSMP proteins from Pinctada, Mytilus & other sp.	Deamidation
P501	23	[Tridacna_crocea]_TRINITY_ DN245948_c1_g1_i1.p1	*Tridacna crocea*	Coiled-coil domain-containing protein	Coil					5.68	2	2	85.93	Myosin-J heavy chain-like & coiled-coil domain-containing proteins from other molluscs	Deamidation
P501	24	[Tridacna_crocea]_TRINITY_ DN230191_c0_g1_i1.p1	*Tridacna crocea*	Uncharacterized	Disordered					11.18	3	3	62.06	Spidroin-1-like proteins from other molluscs	Oxidation, deamidation
Blank		[Tridacna_crocea]_TRINITY_ DN238779_c53_g1_i1.	*Tridacna crocea*	Actin		7.69	3	2	111.06						
		[Sinotaia purificata] GIUB01079209.1.p2	*Sinotaia purificata*	Histone		15.00	2	2	78.02						

Table 2. List of proteins identified in archaeological samples. Peaks Studio 11 software was used together with an in-house molluscan protein database to identify sequences related to certain species (Peptide FDR 1%, protein scores −10lgP ≥ 30, unique peptides ≥ 2). For the second round of search, we narrowed down the database to *Tridacna* sp. proteins, as these were dominating the results of the first search (same parameters as above except protein scores were set to a higher value of −10lgP ≥ 40). The table reports the name of the identified sequence and the species of origin; protein classification and presence of domains (assessed by InterPro), protein coverage in archaeological samples, number of peptides (total and unique) as well as protein identification scores (-10lgP) using both databases; BlastP search results and observed PTMs are given in the last two columns.

Results

Using the Molluscan Protein database, six proteins were identified in sample PALTO499, all of them belonging to *Tridacna crocea*. One protein sequence was identified in sample PALTO500, also belonging to *T. crocea*. Sample PALTO501 had five protein sequences identified belonging to *T. crocea*. Given that most of the protein hits were related to *Tridacna* sp. proteins, we conducted a second round of searches where we narrowed down the database only to include *Tridacna* proteins. Using this *Tridacna* Protein DB, the coverages (and supporting peptides) increased, and we were able to identify additional proteins, all from *Tridacna crocea*: two extra sequences were identified in sample PALTO499, sixteen more for PALTO500 and ten more for sample PALTO501. In all the three samples, twenty-four different *Tridacna* proteins were identified. All of the sequences were those obtained from translated transcriptomes.

A number of sequences were shared among the different samples. No.1 was the top scoring sequence (based on -lgP value), identified in all of the three samples (in PALTO499 and PALTO501 it was found using both databases, i.e., Molluscan and *Tridacna* protein DBs, while in sample PALTO500 it was identified only when searching peptides with *Tridacna* protein DB). The protein coverage varies from 5% with 6 supporting peptides (in PALTO500) to 24% with 58 supporting peptides (PALTO499). It is an uncharacterized protein that possesses disordered domains (assessed by InterPro), with no significant homology to other known sequences (assessed by BlastP search). Several diagenesis related modification (PTMs) are identified among supporting peptides (some are found in all the three samples, at the same positions), including oxidation, deamidation, pyro-glu from Q, dehydration. Sequences No. 2 and 3 are also found in all the three samples. Coverages range from 5-19% with 2-11 supporting peptides (No.2) and 5-7% with 6-9 supporting peptides (No.3). Both of these sequences are uncharacterized, with disordered domains and with no significant homology to other protein sequences. The supporting peptides also show presence of diagenesis related PTMs, including deamidation, oxidation and pyro-glu from E. Other uncharacterized *Tridacna* proteins include sequences No.: 5, 7, 8, 12, 13, 14, 17, 20, 21, 24. For some of these sequences, supporting peptides are LCD type and many other peptides show diagenesis related PTMs (e.g., deamidation, oxidation). We also identified sequences that are characteristic to mollusc shell proteins. No. 4 is a chitin-binding protein and it was found in all the three archaeological samples. The coverage is up to 6% (44 supporting unique peptides are found in sample PALTO501, 30 in PALTO500 and 11 in PALTO499). The protein possesses chitin-binding, vWFA domains and Thrombospondin type-1 repeat and it is homologous to other molluscan proteins. Sequence No.22 is a BSMP-like protein and was found in PALTO501. It also possesses vWFA and chitin-binding domains and it is homologous to other BSMP mollusc shell proteins. Sequence No.9 is a mucin-like protein and it was found in samples PALTO500 and PALTO501. It possesses chitin-binding and disordered domains and it is homologous to other mucin-like and serine-rich proteins from other molluscs. Other identified *Tridacna* sequences include Pleckstrin-like, Coiled-coil domain-containing and RNA-binding proteins.

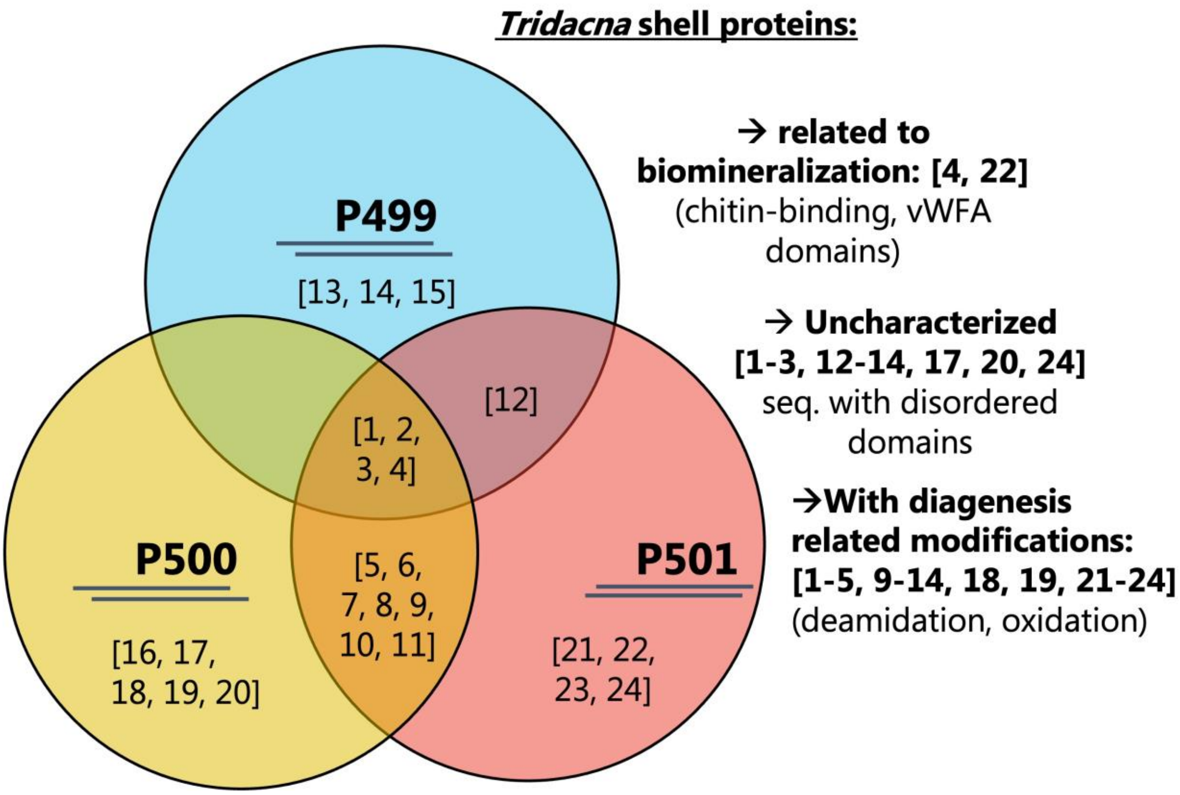

Figure 1. Schematic representation of the results obtained by palaeoproteomic analysis of intracrystalline protein fraction extracted from archaeological beads. The Venn diagram indicates the proteins identified in each of the three samples (PALTO499, PALTO500, PALTO501) and those which are shared. The numbers correspond to sequences provided in Table 2. On the right-hand side, we note sequences that are typical of ancient mollusc shell proteins, i.e., with biomineralizing and disordered domains and sequences that were identified with diagenesis related modifications.

Discussion

The origin of the three archaeological samples can be identified as made of *Tridacna* sp. shells, the giant marine clams. Using a comprehensive Molluscan Protein database, palaeoproteomic analyses allowed us to identify a number of proteins, with most being of *Tridacna* shells. We note that all of our identified sequences were translated from molluscan transcriptomes, highlighting the importance of employing RNA datasets for shell (palaeo)proteomic studies. The identifications were carried out with careful assessment of mollusc shell proteins. Our identifications were based on the analysis of peptides that can be considered 'taxon specific' (we filtered out sequences with low complexity domains, LCDs), a prerequisite for palaeoshellomic analysis (Sakalauskaite et al. 2019). Currently, ShellOmic analyses are not capable of distinguishing at the species level, therefore, we report our findings to genus level - *Tridacna* sp. The customised database that we used for searching peptides from

archaeological samples, contained more than 1.7 million molluscan protein sequences, including many from species that could be potential raw materials for the ornaments, i.e., bivalves such as scallop shells (*Pecten* sp.), the thorny oyster *Spondylus*, oyster shells (*Pinctada* sp.). This is the largest Molluscan protein database used for the identification of ancient molluscan proteins. The peptides obtained from archaeological samples were unequivocally matched to *Tridacna* shell proteins, many of which showed features that are typical among shell proteins (Marin and Luquet 2004; Marin 2020). In particular, we identified proteins that are known to be involved in shell biomineralization, such as chitin-binding and BSMP (Sakalauskaite et al. 2020; Marin 2020). These proteins possess vWFA domains that are characteristic of shell sequences and have been found among other shell proteomes, including *Pecten*, *Mytilus*, *Pinctada* (Marie et al. 2013; Marin et al. 2013; Marie et al. 2017). We also identified mucin-like and RNA binding proteins, but their function in the mineralization process is not well described. Finally, around half of the identified *Tridacna* sequences were uncharacterized proteins and with disordered protein regions. This is a common encounter in shell protein analyses (Kocot et al. 2016; Boskey and Villarreal-Ramirez 2016). Molluscan genomes are complex and for many species they have not been well annotated (Davison and Neiman 2021), therefore, for many, their function is yet unknown. So far, uncharacterized proteins can be considered as a characteristic feature of mollusc shell proteins.

Based on our findings, we argue that the same shell material (species) were used for making the ornaments. Many of the same *Tridacna* proteins were found in all of the three archaeological samples. In addition, some were supported by the same peptide sequences and some of them had degradation related modifications (deamidation, oxidation) at the same positions. Therefore, this confirms that we have isolated endogenous intracrystalline protein sequences. The obtained results mark the oldest molluscan shell proteins recovered and characterised so far.

Data availability

The mass spectrometry proteomics data have been deposited to the ProteomeXchange Consortium via the PRIDE (Perez-Riverol et al., 2022) partner repository with the dataset identifier PXD041082 and 10.6019/PXD041082.

Acknowledgements

JS, MM, MC were supported by DNRF128 grant for the work performed at the University of Copenhagen.

JS acknowledges ASSEMBLE+ program for contributing in obtaining *Spondylus* sequence data and JS would like to thank Daniel Jackson (University of Göttingen) for his input in gathering transcriptome. JS is grateful to Frédéric Marin (UMR CNRS 6282 Biogéosciences, University of Burgundy-Franche-Comté) for valuable insights.

References

Blum, M., Chang, H.-Y., Chuguransky, S., Grego, T., Kandasaamy, S., Mitchell, A., Nuka, G., Paysan-Lafosse, T., Qureshi, M., Raj, S., Richardson, L., Salazar, G.A., Williams, L., Bork, P., Bridge, A., Gough, J., Haft, D.H., Letunic, I., Marchler-Bauer, A., Mi, H., Natale, D.A., Necci, M., Orengo, C.A., Pandurangan, A.P., Rivoire, C., Sigrist, C.J.A., Sillitoe, I., Thanki, N., Thomas, P.D., Tosatto, S.C.E., Wu, C.H., Bateman, A., Finn, R.D., 2021. The InterPro protein families and domains database: 20 years on. Nucleic acids research 49, D344–D354.

Boskey, A.L., Villarreal-Ramirez, E., 2016. Intrinsically disordered proteins and biomineralization. Matrix biology: journal of the International Society for Matrix Biology 52-54, 43–59.

Davison, A., Neiman, M., 2021. Mobilizing molluscan models and genomes in biology. Philosophical transactions of the Royal Society of London. Series B, Biological sciences 376, 20200163.

Galaxy Community, 2022. The Galaxy platform for accessible, reproducible and collaborative biomedical analyses: 2022 update. Nucleic acids research. doi:10.1093/nar/gkac247

Kocot, K.M., Aguilera, F., McDougall, C., Jackson, D.J., Degnan, B.M., 2016. Sea shell diversity and rapidly evolving secretomes: insights into the evolution of biomineralization. Frontiers in zoology 13, 23.

Mackie, M., Rüther, P., Samodova, D., Di Gianvincenzo, F., Granzotto, C., Lyon, D., Peggie, D.A., Howard, H., Harrison, L., Jensen, L.J., Olsen, J.V., Cappellini, E., 2018. Palaeoproteomic profiling of conservation layers on a 14th century Italian wall painting. Angewandte Chemie 57, 7369–7374.

Marie, B., Jackson, D.J., Ramos-Silva, P., Zanella-Cléon, I., Guichard, N., Marin, F., 2013. The shell-forming proteome of *Lottia gigantea* reveals both deep conservations and lineage-specific novelties. The FEBS journal 280, 214–232.

Marie, B., Arivalagan, J., Mathéron, L., Bolbach, G., Berland, S., Marie, A., Marin, F., 2017. Deep conservation of bivalve nacre proteins highlighted by shell matrix proteomics of the *Unionoida Elliptio complanata* and *Villosa lienosa*. Journal of the Royal Society, Interface / the Royal Society 14. doi:10.1098/rsif.2016.0846

Marin, F., 2020. Mollusc shellomes: Past, present and future. Journal of structural biology 212, 107583.

Marin, F., Luquet, G., 2004. Molluscan shell proteins. Comptes rendus. Palevol 3, 469–492.

Marin, F., Marie, B., Hamada, S.B., Ramos-Silva, P., Le Roy, N., Guichard, N., Wolf, S.E., Montagnani, C., Joubert, C., Piquemal, D., Saulnier, D., Gueguen, Y., 2013. “Shellome”: Proteins involved in mollusk shell biomineralization-diversity, functions. In: Watabe, S., Maeyama, K., Nagasawa, H. (Eds.), International Symposium on Pearl Research. Terrapub Tokyo, pp. 149–166.

Perez-Riverol, Y., Bai, J., Bandla, C., García-Seisdedos, D., Hewapathirana, S., Kamatchinathan, S., Kundu, D.J., Prakash, A., Frericks-Zipper, A., Eisenacher, M., Walzer, M., Wang, S., Brazma, A., Vizcaíno, J.A., 2022. The PRIDE database resources in 2022: a hub for mass spectrometry-based proteomics evidences. Nucleic acids research 50, D543–D552.

Rappsilber, J., Mann, M., Ishihama, Y., 2007. Protocol for micro-purification, enrichment, pre-fractionation and storage of peptides for proteomics using StageTips. Nature protocols 2, 1896–1906.

Sakalauskaite, J., Andersen, S.H., Biagi, P., Borrello, M.A., Cocquerez, T., Colonese, A.C., Dal Bello, F., Girod, A., Heumüller, M., Koon, H., Mandili, G., Medana, C., Penkman, K.E., Plasseraud, L., Schlichtherle, H., Taylor, S., Tokarski, C., Thomas, J., Wilson, J., Marin, F., Demarchi, B., 2019. “Palaeoshellomics” reveals the use of freshwater mother-of-pearl in prehistory. eLife 8. doi:10.7554/eLife.45644

Sakalauskaite, J., Plasseraud, L., Thomas, J., Albéric, M., Thoury, M., Perrin, J., Jamme, F., Broussard, C., Demarchi, B., Marin, F., 2020. The shell matrix of the european thorny oyster, *Spondylus gaederopus*: microstructural and molecular characterization. Journal of structural biology 211, 107497.

Takeuchi, T., Fujie, M., Koyanagi, R., Plasseraud, L., Ziegler-Devin, I., Brosse, N., Broussard, C., Satoh, N., Marin, F., 2021. The “Shellome” of the Crocus Clam Tridacna crocea Emphasizes Essential Components of Mollusk Shell Biomineralization. Frontiers in genetics 12, 940.

Welker, F., Ramos-Madrigal, J., Gutenbrunner, P., Mackie, M., Tiwary, S., Rakownikow Jersie-Christensen, R., Chiva, C., Dickinson, M.R., Kuhlwilm, M., Manuel, M. de, Gelabert, P., Martinón-Torres, M., Margvelashvili, A., Arsuaga, J.L., Carbonell, E., Marques-Bonet, T., Penkman, K., Sabidó, E., Cox, J., Olsen, J.V., Lordkipanidze, D., Racimo, F., Lalueza-Fox, C., Bermúdez de Castro, J.M., Willerslev, E., Cappellini, E., 2020. The dental proteome of Homo antecessor. Nature 580, 235–238.

Part II: Infrared Spectroscopy of two Amber bead samples from Ba`ja

Carlos P. Odriozola[1,2], José Ángel Garrido-Cordero[1], José María Martínez-Blanes[3]

[1] Dpto. de Prehistoria y Arqueología, Universidad de Sevilla, Seville, Spain.
[2] UNIARQ, Centro de Arqueologia da Universidade de Lisboa, Lisbon, Portugal.
[3] Instituto de Ciencia de Materiales de Sevilla, Universidad de Sevilla- Consejo Superior de Investigaciones Científicas, Seville, Spain.

Method

We have used FTIR, a well-grounded methodology, to study the origin of 2 amber beads used in the composition of the ornaments' child at the site of Ba`ja .

The beads were tested by Attenuated total Reflectance Fourier Transform Infrared spectroscopy (ATR-FTIR), using a Nicolet IS5 FTIR spectrometer equipped with a ID7 diamond ATR accessory. The data were collected as infrared transmission spectra after scanning each specimen 64 times in the range 4000–400 cm^{-1}, with a resolution of 4 cm^{-1}.

The recorded spectra were then compared to the published reference spectra in order to find matching spectral features or fingerprints that allow for a positive match, thus establishing a probable origin for the tested samples.

Results & discussion

None of the bead's spectra show the so-called Baltic shoulder—an intense absorption peak in the 1160–1150 cm^{-1} range, preceded by a characteristic band between 1250 and 1180 cm^{-1}, typical of ambers from the Baltic [1]. Nor the feature bands of Sicilian amber (simetite) at c. 1241 and 1181 cm^{-1} [2]. Instead, in the C-O stretching region (1300–1000 cm^{-1}), a distinctive COOH C-O stretch vibration at 1228 cm^{-1} together an absorption bands at c. 1152 cm^{-1} were recorded.

The set of recorded spectral features in the analysed samples (fig. 1) are compatible with the spectral features recorded for the amber occurring at Lebanon [3,4].

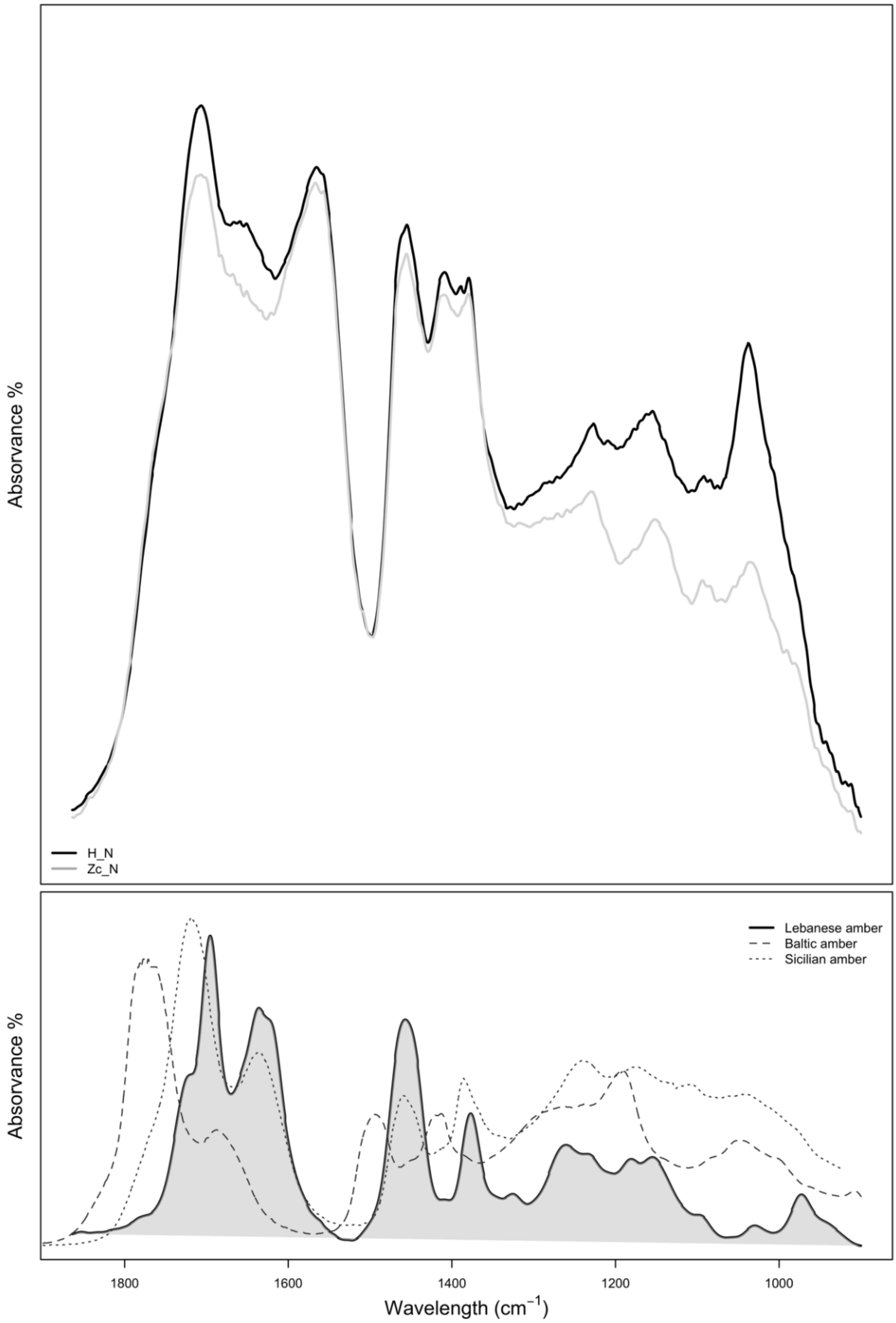

Figure 1. FTIR spectrum in the fingerprint region of the analysed samples compared to standard reference spectrum from different origins (Lebanese amber spectra has been kindly provided by Dr. D. Azar).

Acknowledgements

We are especially grateful to Dr. D. Azar for providing us with the Lebanese amber spectra.
We are thankful for the Junta de Andalucía (Consejería de Economía, Conocimiento, Empresas y Universidad). The analysis of amber beads was funded by the Plan Andaluz de Investigación, Desarrollo e Innovación under contract P20_01080.

References

1. Beck CW, Wilbur E, Meret S, Kossove D, Kermani K. The Infrared Spectra of Amber and the Identification of Baltic Amber. Archaeometry. 1965;8: 96–109.

2. Beck CW, Hartnett HE. Sicilian amber. In: Beck CW, Bouzek J, editors. Proc 2nd Int Conf Amber in Archaeology Liblice 1990. Prague: Czech Academy of Sciences; 1993. pp. 36–47.

3. Kaur S, Stout E, Kaur T, Estridg V. Infrared Spectroscopy of Amber Samples from the Artemision Excavations of 1904/1905. Anatolia Antiqua. 2012;20: 39–43. doi:10.3406/anata.2012.1325

4. Nohra Y, Azar D, Gèze R, Maksoud S, El-Samrani A, Perrichot V. New Jurassic amber outcrops from Lebanon. Terrestrial Arthropod Reviews. 2013;6: 27–51. doi:10.1163/18749836-06021056

Part III: CT scan of two shell beads

Luisa Vigorelli [1,2], Alessandro Re[2]
[1] Electronics and Telecomunication Department, Polytechnic of Torino, Corso Duca degli Abruzzi 24, 10129, Turin, Italy.
[2] Physics Department, University of Torino and INFN, Turin section, Via Pietro Giuria 1, 10125, Turin, Italy.

Micro-CT analysis were carried out at the Physics Department of the University of Turin, where x-ray imaging set-up is installed in a shielded laboratory, consisting of a Hammamatsu Microfocus L8121-03 x-ray source (used focal spot 7µm), a Shad-o-Box 6k HS flat panel detector from Teledyne Dalsa (pixel pitch of 49.5 µm) and a Newport URS50BPP rotating platform on which the sample is placed [ref]. The source and the rotating platform can be moved manually by the operator while the detector is mounted on a mechanical stage for vertical and horizontal movements. All the operation and CT acquisition are conducted remotely from the control room.

The beads:

BJ 03: 12,5 mm h x 5 mm d

BJ 02: 8 mm h x 6 mm d

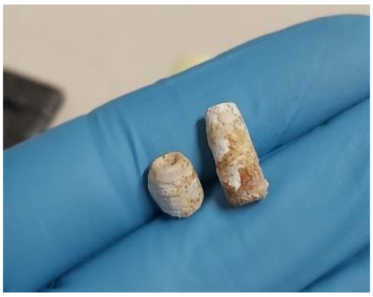

The analysis was performed in order to reach the higher spatial resolution possible, based also on the small bead's dimensions.

CT scan details

Source-Object distance	80 mm
Source-Detector distance	650 mm
Voltage	60 kV
Current	165 uA
Integration time	6 s
Magnification	8×
Voxel	~6 µm
Penumbra	~50 µm

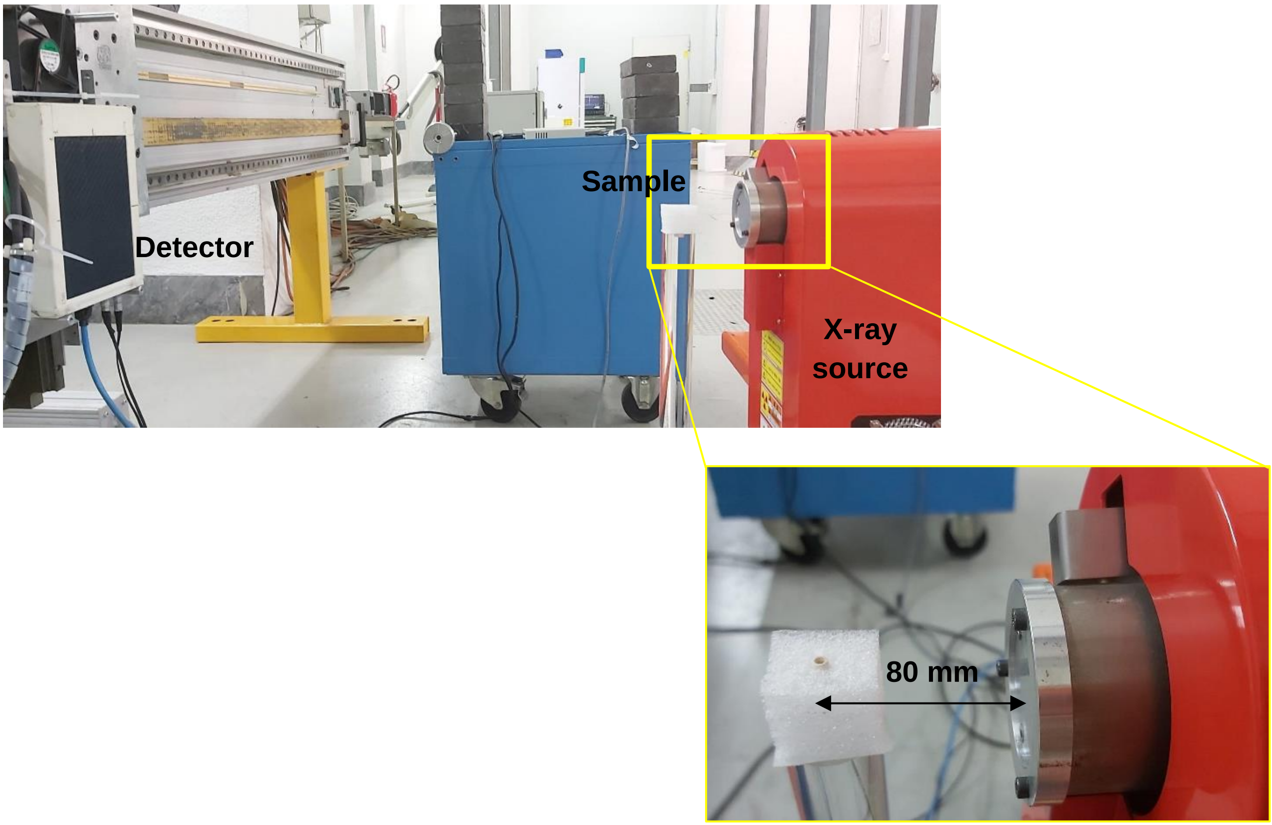

References

Tansella, F.; Vigorelli, L.; Ricchiardi, G.; Re, A.; Bonizzoni, L.; Grassini, S.; Staropoli, M.; Lo Giudice, A. X-ray Computed Tomography Analysis of Historical Woodwind Instruments of the Late Eighteenth Century, J. Imaging 2022, 8, 260. https://doi.org/10.3390/jimaging8100260

Part IV: the morphometric analyses of the disc beads

Hala Alarashi1[1,2]

[1] IMF-CSIC, Barcelona, Spain.
[2] Université Côte d’Azur, CNRS, CEPAM, 06300, Nice, France.

All the elements were 2D scanned using an Epson Perfection 4490 PHOTO scanner. Only those of GN (group of Nice) were measured and morphometrically analyzed. Basic measurements were made manually using an electronic caliper, with values filled automatically on an excel sheet. For the disc beads the diameter, the diameter of perforation, the degree of roundness, and degree of circularities were calculated through Shape Recognition System by applying informatic scripts programmed by the first author using a free online image treatment software (ImageJ Fuji).

The degree of circularity concerns the shape of the outline of the beads. The closer the value is to 1, the more perfect the circle is. The closer the value is to 0.0, the more irregular the outline tends to be. The degree of circularity (parameter "circularity" in ImageJ) is calculated by the formula $4\pi \times \text{area}/(\text{perimeter})^2$.

The degree of roundness examines the shape of the bead independently from its outline (circularity). The closer the value is to 1, the more the general shape corresponds to a disk. The closer the value is to 0.0, the more the bead has an elliptical shape. The degree of roundness of the object (parameter "roundness" in ImageJ) is calculated by the formula: $4\text{x area}/(\pi \times \text{major\_axis}^2)$".

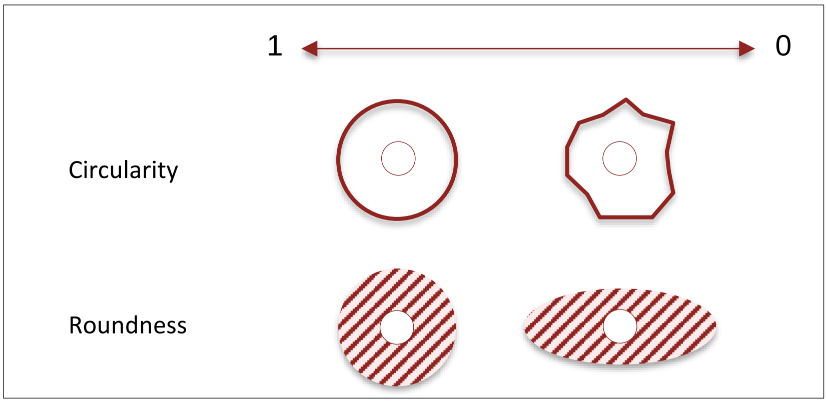

For the analyses of beads, the measurement of the degree of circularity of the outline is useful to evaluate 1) if the finishing stage through polish of the profile was conducted, 2) if it was successful. When the outlines are closer to a circle, it means that the polishing of the profile to give the regular circular shape was conducted and successful. The polish of the surface can however be conducted, yet it might be unsuccessful due to fractures and accidents during the process. This creates irregularity in the outline that are distinguishable from irregularities that are due for not conducting the finishing stage (*i.e.*, the use of beads directly after their shaping into rough discs).

The measurement of the degree of roundness is useful to evaluate if the general shape of the disc bead is circular or on the contrary too oblong, out of round or ellipse in nature. When the roundness is circular, it means that attention and care was kept during the shaping and finishing stages (= regular control of the result). When it is rather oblong, the interpretation can be technical, for example, a prolongated polishing at the same spot.

These interpretations need to be corroborated to experimental work (ongoing), precisely, the polishing of profiles in batch, while stringing the beads together in different ways (in a bead-to bead pattern or separated with smaller leather discs to avoid their contact), using different types of cords or wooding sticks, and adding or not auxiliar agents (water, abrasive). The aim is to test the efficiency of each of the method and see which one provide experimental discs similar in values of roundness and circularity to those of the archaeological ones, but also in terms of microscopic traces.
