# Supplementary material for: Threads of memory: Reviving the ornament of a dead child at the Neolithic village of Ba`ja (Jordan)
Source: PLoS One. 2023 Aug 2;18(8):e0288075. doi: 10.1371/journal.pone.0288075 (PMC10396020; doi:10.1371/journal.pone.0288075)
Supplement: S3 Appendix — PDF with text, four tables and two figures. (PDF) [file pone.0288075.s003.pdf]

Supplementary information 3

Procedure for the reconstruction of the ornament

Hala Alarashi[1,2], Lionel Gourichon[2]

[1] IMF-CSIC, Barcelona, Spain.

[2] Université Côte d'Azur, CNRS, CEPAM, 06300, Nice, France.

The reconstruction of the original shape of the mother-of-pearl ring

The mother-of-pearl ring was damaged when it was discovered. The nacreous was disintegrated into fragile layers, and a small portion had completely vanished. The careful excavation allowed collecting two large fragments: a half and a quarter of the ring. Part of the remaining quarter, the first excavated and the most damaged, was collected into several pieces that were refitted together by Alice Burkhardt, the conservator (Stuttgart academy of Art), who also cleaned and consolidated it. A small portion of the ring remained missing.

Nomenclature and convention of the measurements of the ring are given in Fig 1, while the measurement values are presented in Table 1.

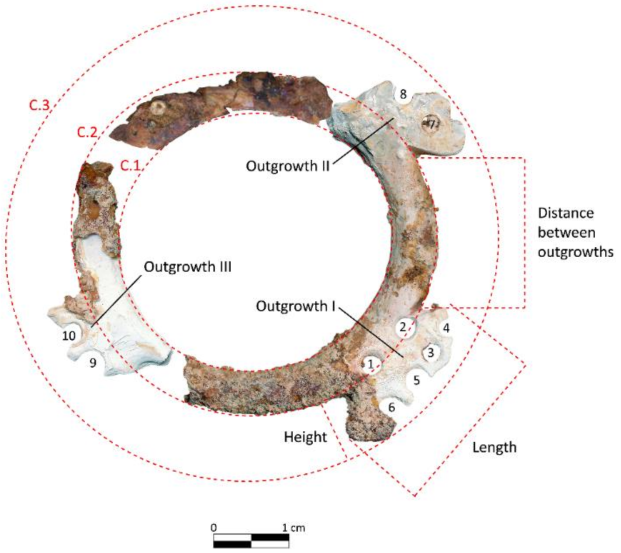

Fig 1 Nomenclature and convention of measurements used for the study. C.1= inner diameter of the ring, C.2=middle diameter, C.3=outer diameter. Each perforation has a number. The engraved parts with perforations are called here "outgrowth" (abbreviated "Outg."). Disc beads were found still stuck to perforation N° 7, 4, 9 and 10.

In the largest fragment of the ring, one almost complete outgrowth (Outg. I) is preserved with six perforations (two complete on the ring area, and four on the outgrowth area, three of them broken), and one broken outgrowth (Outg. II) with two perforations (one complete and one broken). Another small fragment of the ring has a broken outgrowth (Outg. III) with two broken perforations located on the outgrowth area. A total of ten perforations were counted on the preserved fragments of the ring and its outgrowths. The diameter average of the perforations is 2.8mm. The average distance between the two perforations is 3mm. The distance between Outg. I and II is almost the same as the length of Outg. I. Based on the measurements and on the preserved features of the ring, we propose the following hypotheses:

- Not three but four outgrowths: If the three outgrowths measure around 22mm length and 8 to 9mm height each, a fourth outgrowth can be perfectly placed on the opposite side of Outg. I. This makes a ring with four outgrowths distributed symmetrically and separated one from the other by around 25mm. The four outg. pattern is estimated on the basis of other complete rings found at Ba`ja and Basta (cf. *infra*).

- A total of 18 perforations: the three preserved outgrowths bear four equidistant perforations, while one has additional two perforations on the ring part. This part of the ring might have had an additional function than the others. Counting a fourth outgrowth that have probably vanished, the total number of perforations mounts to 18.

- Outgrowths with denticulated edges: the outer edge of Outg. II shows a denticulated shape consisting in a convex line followed by a deep incision that meets near perforation n° 7, as if the artist wanted to mark a visual separation between the areas of perforations within each outgrowth. The shape of the outer edges of the other outgrowths was most likely identical, as hinted by the preserved small edge, near perforation #6 of Outg. I.

Measurements	mm
Outg. I P1	2.7
Outg. I P2	2.83
Outg. I P3	2.38
Outg. I P4	2.38
Outg. I P5	3.26
Outg. I P6	3.11
Outg. II P7	2.53
Outg. II P8	2.55
Outg. III P9	3.87
Outg. III P10	2.46
Distance P1 & P2	3.96
Distance P2 & P3	2.53
Distance P2 & P4	3.13
Distance P1 & P5	3.61
Distance P1 & P6	3.33
Distance P7 & P8	2.77
Distance P9 & P10	1.75
Distance between outg. I & II	20.22
Estimation length outg. I	20.1
Estimation height outg. I	8.9
Estimation height outg. II	8.0
Estimation maximum inner diameter	30.52
Estimation maximum middle diameter	40.68
Estimation maximum outer diameter	60.44
Width ring before outg. II	5.02
Width ring before outg. I	5.58

Table 1. Measurements of the ring.

Previous excavations at Ba`ja have revealed a completely preserved ring (Fig 2, F.no. 30408; Gebel and Hermansen 2001) showing very similar features to those of the ring found in the child's burial. Concretely, it has four outgrowths with equidistant perforations within each; one outgrowth with two additional perforations on the ring portion; denticulated decoration

pattern of the edges. In two lately excavated burials (BJ19-CR17 [CG11] and BJ19-CR28.2 [CG9]), two cross-shaped mother-of-pearl objects, one multiperforated, have also four crossing branches with denticulated edges (Gebel et al. 2020: Fig 34). Denticulated edge is a recurrent motif that characterizes most of the mother-of-pearl items found at the site (Gebel, 1988, Fig 11.6; Gebel et al., 2020, Fig 34 and 42; Alarashi, in press, Fig 5y).

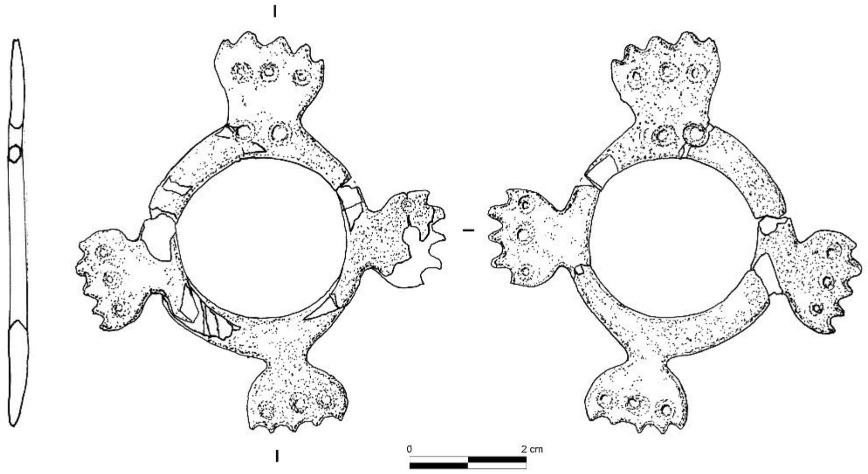

Fig 2. Mother-of-pearl ring ("paillette") – of collective Burial in Area D from Ba`ja. (Photo: H.G.K. Gebel; Drawing: B. Winkler)

Estimations of lengths, number of rows, weight and volume

In order to examine the adequacy of the volume of the ornament when exhibited on an approximately 8-year-old child's, it was necessary to first estimate its length. For this purpose, we based on the metric data directly taken on the beads from the GN (Group of Nice) sample, which represents 65% of the total number of bead elements composing the necklace. The total length of this group was then calculated considering that the beads were all aligned tightly one after the other. Using the dimensions (average, minimum and maximum length, standard deviation) obtained for each type of beads (Table 2), we estimated the total length of the remaining, non-measured beads (GS), assuming that those elements share similar metric proprieties of the GN sample.

The sum of the lengths of both groups was finally calculated as well as the 95% confidence interval. The results were presented per type of beads in Table 3.

To calculate the total length, the following formulas were applied:

- Average estimated total length: total length of measured beads + total estimated length of non-measured beads (sum of N GS * average length of measured beads, per type).
- The minimum of the total length: length measured beads + (N unmeasured beads * average length measured beads - (2*Standard deviation * unmeasured beads).
- The maximum of the total length: length measured beads + (N unmeasured beads * average length measured beads + (2*Standard deviation * unmeasured beads).

Type	Measured beads (GN)						Other beads (GS)				Total Length (estimations)			
	Length					Total length	Estimated total length				N	Mean	Range (95%, mean ±2 s.d.)	
	N	Mean	s.d.	Min.	Max.		N	Mean	Range (95%)					
CDB cal.	1492	1.42	0.347	0.58	3.67	2122.57	772	1098.27	1079.38	1117.17	2264	3220.84	3201.95	3239.74
TB shell	126	9.34	2.274	4.49	14.24	1177.18	106	990.33	944.44	1036.21	232	2167.51	2121.62	2213.39
TB cal.	46	7.26	1.751	4.63	10.25	334.00	20	145.22	129.87	160.56	66	479.22	463.87	494.56
TB resin	1	9.31	0.000	9.31	9.31	9.31	1	9.31	-	-	2	18.61	18.61	18.61
Conus d.	4	2.31	0.492	1.91	2.98	9.22	0	-	-	-	4	9.22	9.22	9.22
ODB tur.	5	2.16	0.575	1.43	2.82	10.78	0	-	-	-	5	10.78	10.78	10.78
FB	7	6.40	6.404	5.50	7.15	44.83	0	-	-	-	7	44.83	44.83	44.83
DPP	1	29.13	0.000	29.13	29.13	29.13	0	-	-	-	1	29.13	29.13	29.13
SB hem.	2	9.34	2.447	7.61	11.07	18.68	0	-	-	-	2	18.68	18.68	18.68
Dental.	1	6.12	0.000	6.12	6.12	6.12	0	-	-	-	1	6.12	6.12	6.12
**TOTAL**	1685					3761.82	899	2243.12	2202.24	2284.00	2584	6004.94	5924.81	6085.07
Total length (in meters)												6.00	5.92	6.09

Table 3. Estimation of the total length of the ornament based on the metric data obtained from the GN (Group of Nice) beads, and the estimated length calculated for the non-measured beads (GS: Group of Stuttgart). The estimations were first made per type of beads, then the results summed. C=circular; D=disc; B=bead; T=tubular; O=oval; F=flat; DPP=double perforated pendant; S=spherical; s.d.= standard deviation.

The minimal numbers of rows composing the necklace was first estimated based on the number of perforations of the outgrowths of the ring that seems to have been the central piece of the ornament. This was based on the fact that several beads were still connected to the perforations of the ring (and the perforations of the stone pendant found behind the neck of the child, most likely as a buckle sustaining the rows of beads spreading from the ring), and because of the occurrence of many portions of alignments of beads below the left side of the child (the preserve part of the ornament).

For the distribution of rows, we favoured the simplest configuration based on the principle of symmetry and equilibrium, imagining the optimal position of the ornament, when it is spread on the chest (the area of concentration of beads).

The whole volume of the ornament was estimated considering standard measurements of the neck, shoulders, arms, and torso of children of both sexes between the ages of seven and nine years according to the AFNOR (French Standardization Association; Table 3). This gave an estimation of a maximum volume of spreading inside an area (the chest) of 30 x 30 cm.

			Girl			Boy	
Years		7	8	9	7	8	9
Hight		120	126	132	120	126	132
Bust circumference	T	56	58	60	58.3	59.8	62.1
	N	62	64	66	60.2	62	64.6
	C	68	70	72	62.7	64.9	67.7
Shoulder breadth	T	23.1	23.8	24.5			
	N	24.3	25	25.7			
	C	25.5	26.2	26.9			
Back breadth	T	24.8	25.6	26.4	26.5	27.5	28.5
	N	26.3	27.1	27.9	27	28	29
	C	27.8	28.6	29.4	27.6	28.6	29.7
Neck circumference	T	25.4	26	26.6			
	N	26.3	26.9	27.5			
	C	27.2	27.8	28.4			
Shoulder's length	T	9	8.6	10.1	9.4	9.9	10.3
	N	9.5	10	10.5	9.5	10	10.4
	C	10	10.4	10.9	9.7	10.1	10.6
7th cervical vertebra to waist (front)	T	34.3	36.4	38.5			
	N	34.8	36.9	39			
	C	35.3	37.4	39.5			
7th cervical vertebra to waist (back)	T	27	28.2	29.4	28	29.2	30.3
	N	27.4	28.6	29.8	28.4	29.5	30.6
	C	27.8	29	30.2	28.8	29.9	31
Collar size	T	28.6	29.3	30	28.2	28.9	29.7
	N	29.5	30.2	30.9	28.7	29.4	30.2
	C	30.4	31.1	31.8	29.2	30	30.8
Shoulder's inclination (slope)	T	23.1°	22,8°	22,6°			
	N	22,9°	22,6°	22,4°			
	C	22,7°	22,4°	22,2°			

Table 4. Adapted from AFNOR measurement chart (unless otherwise stated in cm) of actual children of both sexes aged seven to nine years. T=thin, N=normal, C=corpulent.

Based on these criteria, we adopted the hypothesis of a number of 10 bead rows arranged in a concentric way and thus of decreasing length. The first three (shortest) rows would have been attached simply to the buckle, while the other 7 would have been connected to both the buckle and the ring.

For each row, the combination of the different bead types (Table 5), and the rhythm of their integration of beads was based on the arrangements observed in the field, *i.e.* the alignments of beads *in situ*, such as the direct contact of red disc beads with the ring, the occurrence of this abundant type in all the rows, and the concentration of tubular *Tridacna* and calcite beads in the central part around the neck. The number of beads of each type and their arrangement within each row were then optimally estimated by testing different combinations and arbitrarily choosing one of them.

Separated rows		CDB calcite	TB shell	TB calcite	Total
Row 1	N	100	24	10	134
	Length	142.19	224.22	71.88	438.29
Row 2	N	110	24	10	144
	Length	156.41	224.22	71.88	452.51
Row 3	N	110	26	10	146
	Length	156.41	242.91	71.88	471.20

Connected rows		Right side				Left side				Total			
		CDB calcite	TB shell	TB calcite	Total	CDB calcite	TB shell	TB calcite	Total	CDB calcite	TB shell	TB calcite	Total
Row 4 connected	N	76	13.5	3	92.5	76	13.5	3	92.5	152	27	6	185
	Length	108.12	126.13	21.563	255.81	108.12	126.13	21.563	255.81	216.24	252.25	43.125	511.62
Row 5 connected	N	91	12.5	3	106.5	91	12.5	3	106.5	182	25	6	213
	Length	129.46	116.78	21.563	267.81	129.46	116.78	21.563	267.81	258.92	233.57	43.125	535.61
Row 6 connected	N	111	12	2.5	125.5	111	12	2.5	125.5	222	24	5	251
	Length	157.91	112.11	17.969	287.99	157.91	112.11	17.969	287.99	315.82	224.22	35.938	575.99
Row 7 connected	N	136	10.5	2.5	149	136	10.5	2.5	149	272	21	5	298
	Length	193.48	93.427	17.969	304.87	193.48	93.427	17.969	304.87	386.96	186.85	35.938	609.75
Row 8 connected	N	156	10.5	2.5	169	156	10.5	2.5	169	312	21	5	338
	Length	221.93	93.427	17.969	333.33	221.93	93.427	17.969	333.33	443.86	186.85	35.938	666.65
Row 9 connected	N	186	10	2.5	198.5	186	10	2.5	198.5	372	20	5	397
	Length	264.61	93.427	17.969	376.01	264.61	93.427	17.969	376.01	529.22	186.85	35.938	752.01
Row 10 connected	N	216	10	2	228	216	10	2	228	432	20	4	456
	Length	307.29	93.427	14.375	415.09	307.29	93.427	14.375	415.09	614.58	186.85	28.75	830.18
**L total necklace**		1599.7	1074.4	237.19	2911.3	1599.7	1074.4	237.19	2911.3	3199.3	2148.8	474.38	**5822.5**

Table 5. Estimations of the number of beads per type to be integrated according to the lengths of the rows (cm). C=circular, D=disc, B=bead, T=tubular, O=oval, F=flat, DPP=double perforated pendant, S=spherical.

Additionally, samples of beads per type were weighed. The results were used to estimate the total weight of the elements that make up the ornament, excluding the strings used in its composition, for which we have no evidence regarding their nature(s) or treatments (e.g., with added ochre). The weight of the mother-of-pearl ring was estimated based on its measurements.

References

Alarashi H. Elements of Ornaments in Non-Burial Contexts – Investigations on Raw-Materials, Production, and Use-Wear. In: Benz M, Gresky J, Purschwitz C, Gebel HG, editors. Death in Ba`ja Sepulchral identities and symbolism in the early Neolithic Community of the Transjordanian Highlands Household and Death in Ba`ja 2. Berlin: ex oriente; 2023. Forthcoming.

Gebel HG. Late Epipalaeolithic and aceramic Neolithic sites in the Petra Area. BAR international series 396 (i). In: Garrard AN, Gebel HG, editors. The prehistory of Jordan. The state of research in 1986. BAR international series 396 (i). Oxford; 1988. pp. 67–100.

Gebel HG, Hermansen BD. LPPNB Ba`ja 2001. A short note. Neo-Lithics. 2001 2/01: 15–20.

Gebel HG, Benz M, Purschwitz C, Bader M, Dermech J, Graf J, et al. Household and Death, 3: Preliminary Results of the 13th Season (Spring 2019) at Late PPNB Ba`ja, Southern Jordan 2020. Neo-Lithics 20 Special issue, Ba`ja 2019 season, interim report. 2020.
