# Supplementary material for: Threads of memory: Reviving the ornament of a dead child at the Neolithic village of Ba`ja (Jordan)
Source: PLoS One. 2023 Aug 2;18(8):e0288075. doi: 10.1371/journal.pone.0288075 (PMC10396020; doi:10.1371/journal.pone.0288075)
Supplement: Attachment — Submitted filename: Baja reviewe.docx [file pone.0288075.s004.docx]

**Adorning the dead child at the Neolithic village of Ba`ja (Jordan): A reassessment of the techno-symbolic behavior of early farming communities in the Levant**

This article presents an important archaeological discovery, an outstanding necklet associate with an 8-year-old child burial from c. 8500 years ago. Such an elaborate grave good is indeed unique, and above all includes two amber beads, material never found before in the Levant in such an early context. The article should be published by PLOS ONE. In 2019 PLOS ONE published another article on an outstanding burial from that same site.

The article starts with a general survey on the important of beads in human cultures. Then it describes the site and the burial. Indeed, the excavation conditions were not ideal, so no map of the grave, the skeleton, and the distribution of the grave good is presented in the article. If such map was made, it should be given in publication. These aspects, however, are covered by Figs 1 and 2, together consisted of 15 fields photos.

The presentation of the data, beads and the Mother-of-Pearl pendant, is impressive. We are not told about “green beads”, “red beads”, but substantial mineralogical research identify the stones. Other aspects are well investigated as well, using use-wear analysis, and presenting the evidences for bead manufacturing at the site. All these technical studies are impressive.

My main problem with this article is on the interpretation level.

1. The title of the article includes: “A reassessment of the techno-symbolic behavior of early farming communities in the Levant”. However, there is no a reassessment of the techno-symbolic behavior of early farming communities. And the geographical term “Levant”, is used here for one site only. This term refers to a large territory: southern Turkey, Cyprus, Syria, Lebanon, Israel, Jorden, the Palestinian Authority and Sinai. Is any of these regions mentioned or discussed? The article does not place the burial in this geographical region. Even nearby Pre-Pottery Neolithic B sites with burials, west of the Jorden, like Jericho, Kefar Hahores, Yiftahel, and others, are not mentioned. This article should have a less pretentious title, such as:An outstanding Neolithic child burial from Baja, Jorden.

2. The article raised the question on line 1177-8:“Considering the production and use of the beads raises the question of whether the necklace existed during the lifetime of the child or whether it was created for the death occasion.”. No answer is given here, but in another section, in line 1199:“believed to have been created to endow a highly distinguished 8-year-old child of the community.” The two different possibilities are not discussed at all. There is a need to present the points that support each possibility, and then the authors preference.

The authors have the right to have their own interpretation, but in the Southern Levant the body will start rotten fast and the burial ceremony cannot be delayed for days. How much time is needed to plan and create such a sophisticated necklet. Or maybe it is not so sophisticated if can be made in a few hours?

3. “a highly distinguished 8-year-old child of the community”. Is it not the highly distinguished status of the family, or the parent?

4. The article suggests that (lines 1203-6) “Its association with a child reaffirms the significant place of children for early farming communities in the Levant and questions their indirect role in stimulating bead production activities, in boosting technologies and aesthetics tastes, especially given that they appear to be the main individuals concerned by body ornamentations in such contexts.” No reference is given to other child burials with beads. Indeed, I do not remember even one continuances Neolithic child burial with beads. So, if there are such burials, they should be cited here. As far as I can say, the statement: “given that they appear to be the main individuals concerned by body ornamentations in such contexts” is simply not truth!

5. The reconstruction of the necklet is impressive, but it is presented in photo 18 without a scale, a basic requirement in the publication of any archaeological object. This is not just a technical matter, as the size of the complete necklet may indicates if indeed it fits a young child, or better fit an adult. A scale should be added.

6. The amulet made of Mother-of-Pearl is rather rear, and only two others are known, one of them came from the == burial in Basta (line ===). We are not informed if beads were found at Basta, and what was the age of the buried population.

7. Two unique Neolithic burials had been uncovered at Baja. Such rich burials ae not known from other Neolithic sites in the Levant. Thus, this is a local phenomenon, maybe reflecting a competition between local families, expressed by extravagant consumption. In any case, these graves cannot help in the reassessment of the techno-symbolic behavior of early farming communities in the Levant.

As written above, the article should be published, but unfounded pretentious assumptions should be removed.
